# Killing by Degradation: Regulation of Apoptosis by the Ubiquitin-Proteasome-System

**DOI:** 10.3390/cells10123465

**Published:** 2021-12-08

**Authors:** Ruqaia Abbas, Sarit Larisch

**Affiliations:** Laboratory of Cell Death and Cancer Research, Biology & Human Biology Departments, Faculty of Natural Sciences, University of Haifa, Haifa 3498838, Israel; rabbas18@campus.haifa.ac.il

**Keywords:** ubiquitin proteasome system, ARTS, Smac, XIAP, cIAP, Bcl-2, Mcl-1, parkin, p53, MDM2

## Abstract

Apoptosis is a cell suicide process that is essential for development, tissue homeostasis and human health. Impaired apoptosis is associated with a variety of human diseases, including neurodegenerative disorders, autoimmunity and cancer. As the levels of pro- and anti-apoptotic proteins can determine the life or death of cells, tight regulation of these proteins is critical. The ubiquitin proteasome system (UPS) is essential for maintaining protein turnover, which can either trigger or inhibit apoptosis. In this review, we will describe the E3 ligases that regulate the levels of pro- and anti-apoptotic proteins and assisting proteins that regulate the levels of these E3 ligases. We will provide examples of apoptotic cell death modulations using the UPS, determined by positive and negative feedback loop reactions. Specifically, we will review how the stability of p53, Bcl-2 family members and IAPs (Inhibitor of Apoptosis proteins) are regulated upon initiation of apoptosis. As increased levels of oncogenes and decreased levels of tumor suppressor proteins can promote tumorigenesis, targeting these pathways offers opportunities to develop novel anti-cancer therapies, which act by recruiting the UPS for the effective and selective killing of cancer cells.

## 1. Apoptosis (Programmed Cell Death)

Apoptosis is a morphologically and mechanistically distinct cell death program that is essential for the elimination of unwanted and damaged cells during development and tissue homeostasis [1,2,3]. Abnormal regulation of this process is associated with a wide variety of human diseases, including immunological and developmental disorders, neurodegeneration and cancer [2,3,4]. Apoptosis is executed by caspases, enzymes that are activated following cleavage from their inactive pro-caspase form [5]. Caspase-dependent cell death is the hallmark of apoptosis. However, several alternative modes of non-apoptotic cell death have been described that do not involve caspases [6,7,8,9,10,11]. Apoptosis is regulated by two major pathways: the extrinsic pathway and the intrinsic (mitochondrial) pathway [1,12]. The extrinsic pathway is initiated when apoptotic-inducing ligands bind to death receptors [13]. The intrinsic pathway is mainly induced by internal apoptotic signals, such as DNA damage, as well as by certain external stimuli, such as nerve growth factor (NGF) withdrawal-induced cell death [14]. There is an essential crosstalk between the extrinsic and intrinsic pathways, for example, via caspase-induced-cleavage of BID. Truncated Bid (tBID) is initially cleaved in the extrinsic pathway by caspase-8 and can amplify the apoptotic signal by translocating to the mitochondria and inducing mitochondrial outer membrane permeabilization (MOMP) [15,16,17]. This in turn leads to the release of Cytochrome c and formation of the apoptosome complex, which activates caspase-9 and, subsequently, downstream effector caspases [5,16,17,18,19]. This initiates a proteolytic cascade, which culminates in the cleavage of substrate proteins, leading to the disassembly of the cell [17]. In living cells, caspases are kept controlled by inhibitors of apoptosis (IAP) proteins. The IAP proteins prevent cell death by binding to and inhibiting active caspases [20]. XIAP (X-linked IAP), is the best studied IAP, which has three Baculovirus IAP Repeat (BIR) domains. XIAP-BIR3 domain binds directly to and inhibits caspases -9, and the linker region between XIAP-BIR1 and BIR2 domains inhibits caspase-3 and -7 [21,22,23]. In contrast, cIAP1 and cIAP2 can bind to but not inhibit caspases [24,25]. Both XIAP and cIAPs contain a RING (Really Interesting New Gene) domain, which enables these proteins to function as E3 ligases [26,27,28,29] (see also IAPs section below). Apoptosis is a highly regulated process with pivotal checkpoints. Cancer cells evade apoptosis by disrupting these checkpoints [30,31,32]. The ubiquitin proteasome system (UPS) plays a critical role in keeping this fine-tuned process in check by tightly regulating the levels of anti- and pro-apoptotic proteins [19,33,34,35,36,37,38,39,40,41,42].

## 2. The Ubiquitin Proteasome System

The Ubiquitin Proteasome System (UPS) is responsible for the regulated degradation of intracellular proteins [43,44,45]. Ubiquitylation is the post-translational conjugation of the 76 amino acid ubiquitin protein, which tags proteins destined for degradation via the 26S proteasome [45,46,47,48,49,50]. Ubiquitylation modulates many cellular processes, including DNA replication, DNA repair, cell viability, transcription and apoptosis [51,52,53,54,55,56,57,58,59,60]. The ubiquitin molecule can form eight different polyubiquitin chains by employing one of its seven lysine residues (K6, K11, K27, K33, K48 or K63) or by the methionine residue at position 1 (M1) [61]. Protein modification can be in the form of a mono-, multi-mono- or poly-ubiquitin chains [58,61,62,63,64,65,66,67]. The K48 and the K63 chains account for 80% of the total linkages in mammalians. The K48 chains are involved in proteasome-dependent degradation [68]. In addition, K11 and K29 chains were also found to promote proteasome-dependent degradation [61,69]. In contrast, the levels of K63 chains do not change during proteasome inhibition [70,71,72,73,74,75]. K63 promotes proteasome-independent pathways, such as inflammatory signal transduction, autophagy, endocytosis and DNA repair [76,77]. The ubiquitylation cascade requires a ubiquitin activating enzyme (E1), ubiquitin conjugating enzymes (E2) and the ubiquitin ligases (E3) [78]. The E1 enzyme forms an ATP-dependent thioester linkage with the carboxyl-terminus of ubiquitin; E1 is not substrate-specific [79]. The E2 enzyme receives the activated ubiquitin from E1, which in turn transfers the ubiquitin to the E3 ligase [80,81]. Different E2 enzymes can regulate a single E3 ligase [80,81]. The E3 ligases are substrate-specific and are essential for the final transfer of the activated ubiquitin from the E2 enzyme to the lysine residue onto the target protein [80,81,82].

### E3 Ligases

E3 ligases are the largest and most studied group of the UPS; they can be classified into three major groups. The RING E3 ligase family is the largest, containing 600 members. The HECT family, homologous to Human Papilloma virus E6 Carboxyl Terminal domain, contains 28 members and the RBR (RING between RING fingers) E3 ligase family contains 18 members [83,84,85,86,87]. Many studies have shown that E3 ligases play an important role in oncogenesis by mediating chemo-resistance [88,89,90,91,92,93,94,95,96,97,98,99,100,101]. Hence, any disruption in regulating protein homeostasis via the UPS will result in carcinogenesis and chemo-resistance [102,103,104,105,106,107,108].

The RING E3 ligase family are characterized by the presence of a zinc-binding domain called RING or by a U-box domain [19,109]. The U-box domain is structurally similar to the RING domain but does not contain zinc [110]. The RING E3 ligases do not bind to the ubiquitin itself but rather bind the E2 enzymes carrying activated ubiquitin via the Zn^+2^ within the RING domain [19]. RING E3 ligases can function as monomers, homodimers or heterodimers [111]. The U-box domains can work as either monomers or homodimers [111]. Some RING E3 ligases consist of multiple subunits, such as cullin-RING ligase. Cullins are a family of scaffold proteins that have crucial roles in post-translational modification of proteins involving ubiquitin [112,113]. The cullin RING ligases are composed of cullin as a scaffold proteins that binds a RING-box at its N-terminus, an adaptor protein and a substrate receptor at its C-terminus [112].

The HECT family of E3 ligases acts as an intermediate between the E2 enzyme and target proteins. In these E3 ligases, the ubiquitin is transferred to the cysteine residue of the HECT domain, which is then transferred to the target proteins [84,85].

The RBR E3 ligases are known to have two RING domains (RING1 and RING2) that are linked by an “In-between RING” (IBR) domain [114,115]. Similar to HECT E3 ligases, the RBR E3s act as an intermediate between the E2 enzymes and their substrates [19]. The RING1 binds to the ubiquitin-charged E2 enzyme and allows the transfer of ubiquitin from the E2 enzyme to the cysteine residue of RING2, which in turn transfers it to the target protein [19,114,115].

## 3. The Regulation of Pro- and Anti-Apoptotic Protein by the UPS

The Ubiquitin Proteasome system (UPS) regulates apoptosis by targeting proteins, which are important for executing the apoptotic process. Modulation of apoptosis by the UPS can either initiate or inhibit apoptosis [44,116,117]. The role of ubiquitin in cell death was first reported for the non-apoptotic death of muscle cells during insect metamorphosis [118]. As insects transition from the larval to the pupal stage, the steroid hormone ecdysone triggers the elimination of many larval tissues [119]. The degradation of larval intersegmental muscles was one of the first descriptions of developmental programmed cell death [120]. Ironically, the exact mechanism underlying larval muscle histolysis is still unclear. In this review, we will focus on the regulation of apoptotic proteins by the UPS.

### 3.1. p53

**Figure 1:**
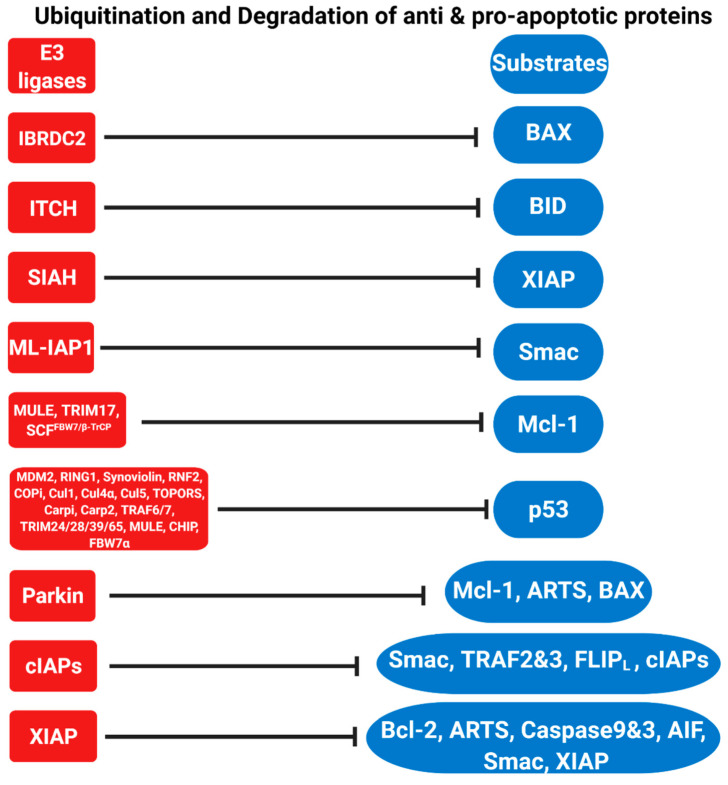
E3 ligases (red) regulate the apoptotic pathway by inducing the degradation of pro- and anti-apoptotic proteins (substrates in blue) via the ubiquitin proteasome system (UPS).

**Figure 2:**
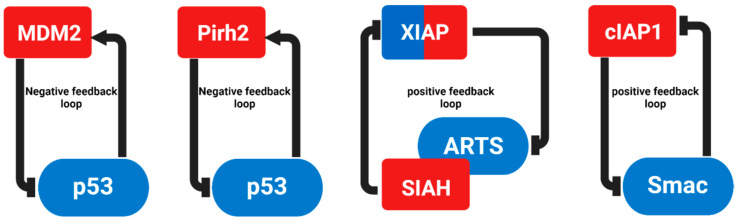
Each of these feedback loops consists of proteins whose levels and function are influenced by the activation or inhibition of their E3 ligase. Arrows indicate stimulatory interactions, whereas horizontal bars denote inhibitory influences.

*p53* is the most intensively studied gene in molecular oncology [121,122]. Nearly 50% of all human tumors have mutations in the p53 gene [122,123,124]. In some cases, the mutated p53 protein is stabilized by evading proteasome-dependent degradation [125]. Since abnormalities in p53 levels can cause tumorigenesis, UPS-mediated degradation is important to keep the levels of p53 in check [126,127,128,129,130,131]. p53 is a major transcription factor that controls a variety of cellular processes, such as growth arrest, DNA repair, senescence and apoptosis [132,133]. In viable cells, p53 is present at low levels and has a short half-life of approximately 5 to 20 min [134,135,136,137,138]. However, following stress signals, including DNA damage, the half-life of p53 increases several-fold through inhibition of its E3 ligases, causing p53 protein accumulation [136,137,138,139,140]. So far, 20 E3 ligases that regulate p53 levels via the UPS have been identified (Table 1, Figure 1). The onco-protein MDM2 is the main E3 ligase that mediates p53 ubiquitylation [141,142,143]. The N-terminal part of MDM2 obstructs the transcription function of p53, while its C-terminal part recruits the E2 ubiquitin conjugate enzyme for ubiquitylation and degradation of p53 [144,145,146]. *MDM2* knockout (KO) mice are embryonically lethal due to excessive p53 accumulation [147,148]. This phenotype can be rescued by the inactivation of p53 [147,148]. Notably, p53 and MDM2 operate in a negative feedback loop (Figure 2); p53 induces the expression of MDM2, which in turn promotes the degradation of p53 [133,149,150,151]. Many tumors display high levels of MDM2 and low levels of p53 and thereby manage to evade apoptosis [144].

Regulation of apoptosis by the UPS also involves other proteins that modulate E3 ligase activity and thereby control the levels of pro-apoptotic proteins. MDM2 is an unstable protein that undergoes ubiquitylation and degradation in an autocatalytic manner [152,153]. MDM2 exhibits increased E3 ubiquitin activity and self-ubiquitylation when it forms homo- or hetero-dimers [154,155]. Moreover, MDM2 stability increases when certain proteins bind to its C-terminal RING domain [156,157,158]. For example, MDM2 activity is regulated by MDMX, a protein that shares sequence similarity with MDM2 but lacks the E3 ligase activity [159]. MDMX and MDM2 form heterodimers that enhance the MDM2 E3 ligase activity and reduce MDM2 auto-ubiquitylation and degradation [158,160,161]. MDMX is essential for the suppression of p53 by MDM2, as MDMX KO mice, similar to MDM2 KO mice, are embryonically lethal due to increased p53 activity. Similar to the MDM2 KO phenotype, these mice can be rescued by the co-deletion of p53 [162,163,164]. In addition, the MDM2 RING domain binds the internal ribosome entry site (IRES) region in XIAP mRNA, thereby preventing homodimerization of MDM2 and its autoubiquitylation. This results in increased MDM2 stability and XIAP translation, leading to cell survival [157,165]. Moreover, p53 promotes the expression of the ubiquitin domain-containing 1 (UBTD1) protein, which in turn induces MDM2 degradation through a positive feedback loop mechanism [166]. Although MDM2 is considered the primary E3 ligase of p53, other E3 ligases were shown to control the stability of p53. Some E3 ligases can promote the degradation of p53 independently of MDM2, such as Pirh2, COP1, ARF-BP1, CHIP, TOPORS, Synoviolin and Carps (see Table 1 for a complete list). Three additional E3 ligases, TRIM28, RNF2 and Cul4a, can promote p53 degradation by interacting with MDM2.

p53-induced RING-H2 protein (Pirh2) is an important transcriptional target of p53. Pirh2 can physically interact with p53, inducing its ubiquitylation and degradation independently of MDM2, which initiates a negative feedback loop (Figure 2) [167,168,169]. Various kinds of cancers express high levels of Pirh2, which is associated with poor prognosis and survival rate [169,170,171]. Using hepatocellular carcinoma samples, the SCYL1-binding protein 1 (SCYL1BP1) was found to promote Pirh2 degradation and thereby restore p53 levels [172]. The COP1 protein contains an N-terminal RING finger and can ubiquitylate p53 independently of MDM2 and Pirh2 under stressed and unstressed conditions [173]. However, COP1^hypo/−^ mice do not exhibit any dysregulation in p53 levels, suggesting that COP1 does not play a role in regulating the levels of p53 [174]. ARF-BP1 (HUWE1, MULE) is a HECT E3 ligase that can also regulate p53 levels independently of MDM2 [175]. Silencing of the ARF-BP1 expression by RNAi in U2OS cells resulted in p53-dependent apoptosis [175]. The TRIM protein family has an N-terminal RING finger domain and at least one B-box zinc finger domain [176]. TRIM28 can induce p53 ubiquitylation and degradation via its interaction with MDM2 [177]. Yet, silencing TRIM28 caused an increase in the expression of p53 target genes [177,178]. U-box E3 ligases can also induce p53 ubiquitylation and degradation. On example is CHIP (carboxyl terminus of Hsc70-interacting protein), a chaperone-interacting protein that has E3 ligase activity and is responsible for the ubiquitylation and degradation of various proteins via its C-terminal U-box [179,180]. CHIP can promote p53 degradation by interacting with Hsc70 [179,180]. CHIP ubiquitylates and degrades both wild-type and mutant p53 via both the proteasome and the lysosome pathways [181,182]. Since p53 regulates various cellular processes, it has been suggested that each E3 ligase is assigned for regulating p53 under certain conditions or in specific tissues or cell types [183].

p53 promotes apoptosis mainly by inducing the transcriptional upregulation of pro-apoptotic proteins, such as the death receptor 5 (DR5), TNFR1 and Fas, which results in the activation of caspase-8 [184,185]. In addition, p53 can induce the activation of pro-apoptotic Bcl-2 family proteins, such as BAX, PUMA, BAD, BID, BAK and NOXA [186,187,188,189,190]. The induction of BID, BAK and BAX promotes the permeabilization of the outer mitochondrial membrane and amplifies the caspase activation process [191,192]. p53 also increases the transcription of the pro-apoptotic XIAP-antagonist, ARTS, which relieves caspases from inhibition by XIAP, leading to the cleavage of BID and MOMP [190,193].

**Table 1:** Ubiquitin E3 ligases targeting p53 for degradation.

E3 Ligase	Type	Effect on p53	Model	Ubiquitination Observed In Vivo/In Vitro	References
MDM2	RING	Degradation	mouse	In vivo and in vitro	[141,142]
ARF-BP1	HECT	Degradation	mouse	In vivo and in vitro	[175]
CHIP	U-box	Degradation	mouse	In vivo and in vitro	[182]
Cop1	RING	Degradation	-	In vivo and in vitro	[173]
Cul1	RING	Degradation	mouse	In vivo and in vitro	[194]
Cul4a	RING	Degradation	mouse	In vivo	[195,196,197]
Cul5	RING	Degradation	-	In vitro	[198]
Synoviolin	RING	Degradation	drosophila	In vivo and in vitro	[199]
TOPORS	RING	Degradation	-	In vivo and in vitro	[200]
Trim24	RING	Degradation	drosophila	In vivo and in vitro	[201]
TRIM28	RING	Degradation	-	In vitro	[202]
TRIM39	RING	Degradation	-	In vitro	[203]
TRIM65	RING	Degradation	-	In vivo	[204]
Carpi	RING	Degradation	-	In vivo	[205]
Carp2	RING	Degradation	-	In vivo	[205]
Pirh2	RING	Degradation	mouse	In vivo and in vitro	[169]
TRAF6	RING	Degradation	mouse	In vivo and in vitro	[206]
TRAF7	RING	Degradation	-	In vitro	[207]
RNF2	RING	Degradation	mouse	In vivo and in vitro	[208]
RING1	RING	Degradation	-	In vivo and in vitro	[209]

### 3.2. BCl-2 Family

Members of the Bcl-2 family of proteins mainly modulate the intrinsic (mitochondrial) pathway [210,211]. This family contains both pro- and anti-apoptotic proteins that share up to four conserved Bcl-2 homology (BH) domains and form complexes by binding to their common BH3 domains [212,213]. The balance between pro- and anti-apoptotic proteins of the Bcl-2 family determines the sensitivity of cells to apoptotic stimuli [214]. The Bcl-2 family can be divided into three subgroups: anti-apoptotic (Bcl-2, Bcl-xL, Bcl-w, Mcl-1, A1 and Bcl-B), pro-apoptotic (BAX and BAK), and pro-apoptotic BH3-only proteins (Bim, Bad, tBid, Bmf, Bik, Noxa, Puma and Hrk) [211,214]. Bcl-2 itself is a key cell death regulator that inhibits cell death [215]. Many cancers are characterized by high levels of Bcl-2 [212,213,216,217,218]. In apoptotic cells, Bcl-2 levels decrease as a result of their degradation by the UPS [36,219,220,221]. XIAP serves as the E3 ligase for Bcl-2 (Figure 1) [36]. Upon apoptotic stimuli, Bcl-2 is brought into close proximity to XIAP by ARTS, which serves as a scaffold protein. The formation of a ternary complex between Bcl-2, ARTS and XIAP enables Bcl-2 ubiquitylation by XIAP and its subsequent degradation by the proteasome [36].

The myeloid cell leukemia 1 (Mcl-1) protein is involved in attenuating the apoptotic response upon DNA damage, growth factor withdrawal and adenoviral infection [222]. Proteasome-mediated degradation of Mcl-1 is crucial for its proper function [223,224]. The first identified E3 ligase of Mcl-1 was the “Mcl-1 ubiquitin E3 ligase” (MULE/ARF-BP1). MULE contains a BH3 domain that assists in binding to Mcl-1 but not to Bcl-2 or Bcl-xL [222,225]. Another E3 ligase that regulates Mcl-1 during neuronal apoptosis is Tripartite Motif-containing protein 17 (TRIM17) [226]. Parkin is yet another E3 ligase that is involved in the degradation of Mcl-1 during mitochondrial depolarization [227]. Furthermore, SCF^β-TrCP^ and SCF^FBW7^ are two additional E3 ligases of Mcl-1 that belong to the SCF family (Skp1, Cullin, F-box complex) [228,229,230]. Finally, APC/C^Cdc20^ (APC/C complexed with substrate recognition adapter Cdc20) was proposed to mediate Mcl-1 ubiquitylation during mitotic arrest (Figure 1) [231,232]. This long list of E3 ligases targeting Mcl-1 illustrates the extent and complexity that is devoted to the Ub-mediated degradation of this protein.

BID is an important pro-apoptotic Bcl-2 family protein. In response to apoptotic stimuli, BID is cleaved to generate tBID, which in turn binds to BAX and BAK and promotes MOMP [5,16,17,18,19]. tBID connects the extrinsic apoptotic signaling pathway with the intrinsic pathway, thus allowing the amplification of external apoptotic stimuli [5,16,17,18,19]. BID levels are also tightly regulated by E3 ligases, such as the Itchy homolog E3 ligase (ITCH/AIP4). ITCH/AIP4 specifically binds to and ubiquitylates tBID but not the uncleaved form of BID (Figure 1) [233].

In living cells, Bax is mainly present in the cytosol. However, in response to apoptotic stimuli, Bax is activated by undergoing a conformational change that causes translocation to the mitochondrial outer membrane (MOM) [234,235,236]. There, BAX binds to BAK to initiate MOMP, allowing the release of pro-apoptotic proteins. These include Smac/Diablo (Smac) and Cytochrome c (Cyto c), which normally reside in the mitochondrial inner membrane space (IMS) [237]. Since activation of Bax and its translocation to the MOM act as a major regulatory checkpoint in apoptosis, Bax protein levels are tightly controlled by the UPS [238,239,240]. The in-between RING (IBR) domain containing 2 (IBRDC2) E3 ligase induces the degradation of BAX in response to p53-mediated apoptosis [241]. IBRDC2 is highly specific for BAX, as it does not bind to BAK, PUMA or NOXA [241]. Johnson et al. identified Parkin as another E3 ligase that can ubiquitylate BAX, thereby limiting the mitochondrial pool of BAX under non-apoptotic and stress conditions (Figure 1) [242].

### 3.3. Inhibitor of Apoptosis (IAPs) Proteins and IAPs-Antagonists

In viable cells, the activity of caspases is inhibited by IAPs [20,24]. The IAP family consists of eight members in mammals: XIAP, cIAP1, cIAP2, ML-IAP, NAIP, ILP2, Survivin and Bruce [243]. IAPs contain between one to three Baculoviral IAP repeat (BIR) domains, which act as a protein–protein interaction domain [5,17,25]. In addition, XIAP, cIAP1, cIAP2, ML-IAP and ILP2 possess a ubiquitin-associated (UBA) domain that assists in the binding of poly-ubiquitin conjugates and a RING domain responsible for their E3 ligase activity [26,27,28,29]. XIAP is the most potent member of the IAP family in terms of its ability to directly inhibit caspases and suppress apoptosis [244]. cIAPs interact with TNF-associated factors (TRAFs) to hinder the formation of pro-apoptotic signaling complexes in the extrinsic apoptotic pathway initiated by TNFR [245,246,247,248]. It is noteworthy that cIAPs mediate cell survival through both canonical and non-canonical NF-κB signaling [247,249,250,251,252,253,254]. Numerous tumors overexpress XIAP and cIAPs, which enables cancer cells to escape apoptosis [255,256]. Accordingly, both XIAP and cIAPs have become promising targets for cancer therapy [257,258,259,260]. In cells undergoing apoptosis, IAP-antagonists inactivate IAPs, leading to de-repression of active caspases [261,262]. The mammalian IAP-antagonist proteins are Smac, Omi/HtrA2, XAF1 (XIAP-associated factor 1) and ARTS [37,257,263,264,265,266,267,268]. Smac and Omi/HtrA2 contain a conserved four-amino acid domain (AVPI/F), which was first described in the *Drosophila* IAP-antagonists Reaper, Hid and Grim and termed IBM (IAP-binding motif) [269,270,271,272]. Genetic and biochemical characterization of *reaper*, *hid*, *grim* and *diap1* (*Drosophila* IAP1) provided the first evidence for the critical physiological role of IAPs and their antagonists in regulating apoptosis [271,273,274,275,276]. Smac resides in the inner-membrane space of mitochondria [263,264,277]. Upon apoptotic induction, Smac and Cytochrome C (Cyto c) are released into the cytosol [263]. Cyto c, together with APAF-1 and pro-caspase-9, then form the “apoptosome” complex, which cleaves and activates caspase-9 [278]. Smac binds to the caspase-9 pocket in the BIR3 domain of XIAP via its IBM, resulting in the release of XIAP-bound-caspases [263,279,280,281]. Smac binds to cIAP1, cIAP2 and XIAP, yet it only induces ubiquitylation and degradation of cIAPs but not XIAP (Figure 2) [282,283]. ARTS (Sept4_i2) is a splice variant derived from the Sept4 (Septin 4) gene and the only splice variant that functions as a pro-apoptotic protein [284]. ARTS is a tumor-suppressor protein that is localized at the Mitochondrial outer membrane (MOM) [193]. Upon apoptotic stimuli, ARTS rapidly translocates to the cytosol in a caspase-independent manner, where it binds and antagonizes XIAP [37,193]. Furthermore, the translocation of ARTS from the MOM to the cytosol precedes MOMP and the release of Cyto c and Smac and is required for it [36,193]. The localization of ARTS at the MOM facilitates its rapid translocation to the cytosol and binding to XIAP within minutes following apoptotic stimuli [193]. The direct binding of ARTS to XIAP enables de-repression of caspases, which are required for MOMP, and the subsequent release of Cyto c and Smac [36,193,279,285,286,287,288,289,290]. Under steady state conditions, XIAP inhibits unwanted apoptosis by promoting the degradation of active caspase-9, -3 and the apoptosis-inducing factor (AIF) through the UPS (Figure 1) [55,291,292,293]. Furthermore, XIAP attenuates apoptosis by degrading two substrates—both its antagonists Smac and ARTS (Figure 1) [34,294,295]. ARTS levels are also regulated by the E3 ligase Parkin (Figure 1) [296]. ARTS acts as the physiological antagonist of XIAP as Sept4/ARTS KO mice exhibit high levels of XIAP [33,35,37,297,298,299]. Consequently, these ARTS-deficient mice develop various spontaneous cancers— mainly lymphoma and leukemia [299]. Importantly, ARTS is also required for the ubiquitylation and degradation of XIAP by another E3 ligase, seven in absentia homolog (SIAH) (Figure 1 and Figure 2) [298]. cIAP1 E3 ligase activity affects the levels of Smac (in a negative feedback manner) (Figure 2), in addition to TRAF2, TRAF3 and FLIP_L_ (Figure 1) [249,293,300,301,302,303,304]. ML-IAP is another E3-ligase that regulates the levels of Smac, and it has been proposed that this may serve to inhibit apoptosis and promote drug resistance in melanoma cells (Figure 1) [29,305]. Finally, the HECT (homologous to E6-AP carboxyl terminus) family E3 ubiquitin ligase AREL1 can inhibit apoptosis by ubiquitylating all three IAP antagonists Smac, HtrA2 and ARTS [306]. Therefore, UPS-mediated protein degradation plays a critical and complex role for both the function and regulation of IAPs and their antagonists.

## 4. Targeting the UPS for Apoptosis-Induced Cancer Therapy

Cancer cells engage the UPS for degrading pro-apoptotic proteins [307,308]. Hence, evading apoptosis by tipping the balance between pro- and anti-apoptotic proteins may allow initiation of tumorigenesis [4,309,310,311,312]. Intensive efforts are being made to use the UPS for killing cancer cells. The original strategy was to develop general proteasome inhibitors for the treatment of multiple myeloma, which lead to several FDA-approved drugs for the treatment of multiple myeloma (see below) [313,314,315]. This success has encouraged additional efforts to develop new cancer therapeutics by targeting UPS-mediated protein degradation [316,317,318].

### 4.1. Proteasome Inhibitors

At this time, three proteasome inhibitors have been approved by the FDA for the treatment of multiple myeloma: Bortezomib (Velcade, PS341), its second-generation derivative Carfilzomib (Kyprolis) and Ixazomib (MLN9708, Ninlaro) [313,314]. Bortezomib was the first proteasome inhibitor to be approved by the FDA in 2003 [319,320,321,322]. Bortezomib inhibits the 20S proteasome subunit, affecting several vital cellular pathways, including the NF-κB signaling, thereby promoting apoptosis [320,323]. Moreover, Bortezomib increases the levels of the pro-apoptotic protein NOXA [324]. Carfilzomib was the second proteasome inhibitor to be approved by the FDA in 2012 [325,326]. Carfilzomib is a more potent inhibitor of the proteasome when compared to Bortezomib, and it is effective against Bortezomib-resistant multiple myeloma [325,326]. Carfilzomib is thought to induce apoptosis by increasing NOXA levels, which results in the activation of capase-3 and -7 [327]. Unfortunately, despite its effectiveness, Carfilzomib shows dose-limiting toxicities [112].

Ixazomib is the first orally administered proteasome inhibitor; it is as effective as Bortezomib, with respect to inhibiting the proteasome, but has better pharmacokinetic properties. Ixazomib was approved by the FDA and is administered in combination with lenalidomide and dexamethasone in patients with relapsed and refractory myeloma [328,329]. Significantly, Ixazomib can induce apoptosis in Bortezomib-resistant multiple myeloma patients [330]. Delanzomib is another orally administered proteasome inhibitor that inhibits NF-κB signaling and can promote apoptosis in multiple myeloma [331]. Delanzomib is also more effective than Bortezomib in treating normal human epithelial bone marrow progenitor and bone marrow-derived stromal cancer cells [331]. Despite its relative effectiveness, phase II clinical trials with Delanzomib were terminated due to considerable toxicity [332]. Another proteasome inhibitor is Oprozomib, an oral tripeptide epoxyketone. Oprozomib has a longer half-life than Bortezomib and causes activation of caspases-9, -3 and -7 and apoptosis [333,334,335]. Finally, Marizomib is the first natural proteasome inhibitor derived from *Salinosporamide tropica*, a marine actinomycete bacterium [336]. Marizomib causes irreversible inhibition of the 20S proteasome when tested in in vitro and in vivo models [336]. Both Oprozomib and Marizomib underwent clinical trials as a single agent or in combination with other drugs [329]. Despite the relatively high efficacy of general proteasome inhibitors to treat multiple myeloma, their long-term use is limited due to aqcuired resistance towards these compounds [337].

### 4.2. Cancer Therapies Targeting p53 and MDM2 for Degradation

Because of their vital role in maintaining protein turnover, E3 ligases are also complicit in assisting tumorigenesis. Therefore, E3 ligases present promising drug targets for cancer treatments. The inhibition of E3 ligases is supposed to be more target-specific and show lower toxicity compared to general proteasome inhibitors [316].

MDM2 is essential for restricting the levels of p53. Various cancers overexpress MDM2 to hinder the p53-mediated pathway, thus resulting in tumor progression [338,339]. Therefore, MDM2 became an emerging target for developing cancer treatments [316]. The Nutlin small molecules are a family of cis-imidazoline analogs first described as selective and potent inhibitors of MDM2 [340,341]. Nutlins can occupy the binding site of p53 in MDM2 and allow p53 to escape MDM2-mediated ubiquitylation and degradation [340]. Amongst the Nutlin family members, only the enantiomer Nutlin-3a exhibited a potent binding ability to MDM2. Yet, these molecules were not effective enough to be further examined in clinical trials [342]. Resolving the crystal structure of Nutlin-3a facilitated the discovery of better MDM2-p53 inhibitors, such as RG7112, which is currently in phase I clinical trials [343,344,345]. Other small molecules that target the interaction between MDM2 and p53 have been developed and are under various stages of clinical trials. These include AMG-232, APG-115, BI-907828, CGM097, RG7388, DS-3032b and HDM201 [346,347,348].

### 4.3. Cancer Therapies Targeting Bcl-2 Family Proteins for Degradation

Several compounds that target various Bcl-2 family members have been developed. Of notable success is the Bcl-2-specific inhibitor Venetoclax (ABT-199), which has been FDA-approved for the treatment of chronic lymphocytic leukemia (CLL), small lymphocytic lymphoma (SLL), and acute myeloid leukemia (AML) [349,350,351,352,353].

More recently, intense efforts have been made to develop drugs that mediate protein degradation, rather than just binding and inhibiting target protein activity. A major advantage of this approach is the ability to target what were classically considered “non-druggable proteins” [354,355,356,357]. Proteolysis Targeting Chimeras (PROTACs) are small molecules that can promote the ubiquitylation of target proteins by directing specific E3 ligases to specific substrates [318,358,359]. Mechanistically, PROTACs form a ternary complex by binding the protein of interest and an E3 ligase, resulting in the ubiquitylation and degradation of the target protein [359]. For example, Bromodomain Extra-Terminal chimeric molecules (BET-PROTACs), such as ARV-825 and ARV-771, are capable of binding specific target proteins and inducing their ubiquitylation and degradation [360]. Recently, Bcl-xL, Mcl-1 and Bcl-2 PROTACs have been developed [361,362,363,364,365]. DT2216 is the most promising Bcl-xL-specific PROTAC and brings Bcl-xL to the Von Hippel-Lindau (VHL) E3 ligase for degradation by the proteasome [362]. DT2216 is derived from the ABT-263 and showed higher selectivity to kill Bcl-xL-dependent cancer cells than ABT-263 [361,362]. The dMcl1-2 and C3 PROTACs induce Mcl-1 degradation by bringing it into close proximity to cereblon (CRBN) cullin-4A RING E3 ligases [364,365]. In addition, C5 PROTAC was shown to potently and selectively induce the ubiquitylation and proteasomal degradation of Bcl-2 [364].

### 4.4. Cancer Therapies Targeting IAPs for Degradation

IAPs are overexpressed in various tumors, making them attractive drug targets for cancer therapy [366,367,368]. In the past few years, efforts have been made to target IAPs, and specifically XIAP, by small molecules “Smac-mimetics” (SMs) [280,281,369,370,371,372,373]. SMs are small molecules that were based on the evolutionary conserved tetra-peptide IAP-Binding Motif (IBM, AVPI/F). This motif was originally observed in the *Drosophila* IAP-antagonists Reaper, Hid, Grim and is also found in the mammalian IAP-antagonists Smac and Omi [270,271,273,274,280,370]. There are two classes of SMs: monovalent, which contain one AVPI binding motif and bivalent, which contain two AVPI binding motifs and are more potent than the monovalent [371,374]. SMs were originally designed to target and inhibit XIAP [269,372,375,376,377]. Although SMs can bind XIAP, they are not very effective in degrading it [247,373,374,378]. On the other hand, SMs efficiently promote the degradation of cIAPs via the UPS [283,379,380]. The SM-mediated inhibition of cIAPs causes apoptosis through the inhibition of NF-κB signaling [250,251,380,381,382,383,384,385,386,387]. SM130 and SM114 primarily target cIAPs for degradation but have reduced affinity towards XIAP [373]. TL32711 (Birinapant) is a bivalent molecule that works particularly well against cIAP1 and is well tolerated at doses that sustain target inhibition [388,389,390,391]. Unfortunately, most cancer cell lines are resistant to SMs [383,392,393]. Therefore, combinations with other anti-cancer drugs are being explored in an effort to overcome resistance [394,395,396]. Historically, SMs were developed to target XIAP, but until recently, no potent XIAP-only inhibitors were available [34,356]. Mamriev et al. reported of small molecule ARTS-mimetics that can bind XIAP and promote its degradation via the UPS [397]. This compound was identified in a virtual screen for small molecules with the highest docking affinity to the specific and unique binding site of ARTS within the BIR3 domain of XIAP [397]. These small-molecule ARTS-mimetics can induce apoptosis in a wide range of cancer cells but not in healthy PBMC (Peripheral Blood Mononuclear Cells). ARTS-mimetic small molecules bind specifically to XIAP, but not cIAP1, and promote the degradation of both XIAP and Bcl-2 through the UPS [397]. ARTS-mimetics act as PROTACs by bringing the E3 ligase XIAP to its target Bcl-2, thereby inducing the degradation of both these proteins [397]. ARTS-mimetics provide a promising novel platform for developing highly specific and potent anti-cancer drugs by targeting XIAP-and Bcl-2 for degradation [397].

Another strategy to target IAPs for degradation is by a series of chimeric molecules termed specific and non-genetic inhibitor of apoptosis protein (IAP)-dependent protein erasers (SNIPERs). SNIPERs consist of three distinct parts: a target protein ligand, an E3 ligase ligand and a linker between them [398,399,400,401]. They were shown to recruit IAPs and promote their targeted protein degradation [398,399,402,403]. Unlike the traditional PROTACs, SNIPERs induce simultaneous degradation of IAPs, such as cIAP1 and XIAP, along with their target proteins [399,404]. Although PROTACs and SNIPERs exhibited promising results in degrading target proteins, there are still some challenges to overcome before these compounds can be used in the clinic [401]. For example, most PROTACs do not obey Lipinski’s rule of five (RO5) because of their relatively high molecular weight [405]. In addition, PROTAC’s toxicity, bioavailability, distribution and metabolism still need to be determined [401]. For now, two PROTACs have entered phase I/II clinical trials— the PROTAC ARV-110 for the treatment of prostate cancer and ARV-741 for the treatment of breast cancer [401].

## 5. Future Directions and Challenges

The UPS is considered to be a major target for developing novel types of anti-cancer drugs. This was initiated with the use of Proteasome inhibitors, which were proven to be effective for patients with hematological malignancies (Mantle cell lymphoma and multiple myeloma) [406]. However, the efficiency of proteasome inhibitors is seriously compromised due to innate and acquired drug resistance [407,408]. Numerous studies have helped uncover the pathways responsible for drug resistance, making it easier to predict which patients can benefit from specific proteasome inhibitor therapy [409,410,411,412]. One of the approaches proposed to overcome drug resistance is combination therapy [406,412]. This might also help with treating malignancies, which present a limited response to proteasome inhibitors. In many cases, upregulation of E3 ligases is responsible for drug resistance [58,192,413]. Fortunately, many E3 ligases, such as cIAP, XIAP, MDM2 and others, have become popular targets for drug development, including specific small molecules, targeting E3-ligases. Nevertheless, there are still challenges to overcome, including the vast diversity of E3 ligases and the fact that E3 ligases can have various different substrates, including tumor suppressors and oncogenes [414]. The combination of small molecules with other cancer therapies showed better efficacy than monotherapies [414]. Besides small molecules, the protein-targeting chimeras (PROTACs) were developed to overcome drug resistance via hijacking the UPS mechanism. While PROTACs are showing promise in providing a novel approach to overcome drug resistance, many challenges await to be resolved regarding their drug design and possible clinical applications.

In summary, the UPS plays a major role in regulating key apoptotic proteins. As many as 20 E3 ligases alone are known to control the levels of the p53. This illustrates the importance of regulated protein degradation and governing the activity of this major tumor-suppressor protein. High levels of Bcl-2, XIAP and cIAPs are characteristic of many types of cancers and hence make these proteins attractive drug targets [366,367,368,415,416]. Interestingly, far fewer E3 ligases control the levels of these proteins compared to p53. A possible reason for this difference is that these apoptosis-suppressing proteins are regulated by direct binding to neutralizing proteins (such as Bax in the case of Bcl-2) or IAP-antagonists [35,263,417]. Perhaps this regulation through protein–protein interactions can complement any possible limitations resulting from the relatively small number of specific E3 ligases dedicated to these proteins. Degrading the target protein rather than binding and blocking their function has significant advantages; mainly, it reduces the load of the elevated expression of the target protein, which are often inhibitory proteins. Moreover, it can reduce systemic drug concentrations and, hence, possible cytotoxic side effects. Recently, major efforts have been devoted both in academia and by pharma companies to develop therapies that recruit the UPS for promoting apoptosis in cancer cells. Compounds that specifically target proteins for degradation resulting in effective tumor killing may dramatically improve the success of cancer therapy.

## References

[1] Kerr J.F., Wyllie A.H., Currie A.R. (1972). Apoptosis: A basic biological phenomenon with wide-ranging implications in tissue kinetics. Br. J. Cancer.

[2] Meier P., Finch A., Evan G. (2000). Apoptosis in development. Nature.

[3] Fuchs Y., Steller H. (2011). Programmed cell death in animal development and disease. Cell.

[4] Thompson C.B. (1995). Apoptosis in the pathogenesis and treatment of disease. Science.

[5] Donepudi M., Grutter M.G. (2002). Structure and zymogen activation of caspases. Biophys. Chem..

[6] Aram L., Yacobi-Sharon K., Arama E. (2017). CDPs: Caspase-dependent non-lethal cellular processes. Cell Death Differ..

[7] Arama E., Baena-Lopez L.A., Fearnhead H.O. (2021). Non-lethal message from the Holy Land: The first international conference on nonapoptotic roles of apoptotic proteins. FEBS J..

[8] Feinstein-Rotkopf Y., Arama E. (2009). Can’t live without them, can live with them: Roles of caspases during vital cellular processes. Apoptosis.

[9] Fogarty C.E., Bergmann A. (2017). Killers creating new life: Caspases drive apoptosis-induced proliferation in tissue repair and disease. Cell Death Differ..

[10] Baena-Lopez L.A., Arthurton L., Xu D.C., Galasso A. (2018). Non-apoptotic Caspase regulation of stem cell properties. Semin. Cell Dev. Biol..

[11] Kuranaga E., Miura M. (2007). Nonapoptotic functions of caspases: Caspases as regulatory molecules for immunity and cell-fate determination. Trends Cell Biol..

[12] Buttke T.M., Sandstrom P.A. (1994). Oxidative stress as a mediator of apoptosis. Immunol. Today.

[13] Lavrik I., Golks A., Krammer P.H. (2005). Death receptor signaling. J. Cell Sci..

[14] Kristiansen M., Ham J. (2014). Programmed cell death during neuronal development: The sympathetic neuron model. Cell Death Differ..

[15] Luo X., Budihardjo I., Zou H., Slaughter C., Wang X. (1998). Bid, a Bcl2 interacting protein, mediates cytochrome c release from mitochondria in response to activation of cell surface death receptors. Cell.

[16] Li H., Zhu H., Xu C.J., Yuan J. (1998). Cleavage of BID by caspase 8 mediates the mitochondrial damage in the Fas pathway of apoptosis. Cell.

[17] Thornberry N.A., Lazebnik Y. (1998). Caspases: Enemies within. Science.

[18] McComb S., Chan P.K., Guinot A., Hartmannsdottir H., Jenni S., Dobay M.P., Bourquin J.P., Bornhauser B.C. (2019). Efficient apoptosis requires feedback amplification of upstream apoptotic signals by effector caspase-3 or -7. Sci. Adv..

[19] Sharma A., Trivedi A.K. (2020). Regulation of apoptosis by E3 ubiquitin ligases in ubiquitin proteasome system. Cell Biol. Int..

[20] Salvesen G.S., Duckett C.S. (2002). IAP proteins: Blocking the road to death’s door. Nat. Rev. Mol. Cell Biol..

[21] Deveraux Q.L., Reed J.C. (1999). IAP family proteins-suppressors of apoptosis. Genes Dev..

[22] Deveraux Q.L., Leo E., Stennicke H.R., Welsh K., Salvesen G.S., Reed J.C. (1999). Cleavage of human inhibitor of apoptosis protein XIAP results in fragments with distinct specificities for caspases. EMBO J..

[23] Suzuki Y., Nakabayashi Y., Nakata K., Reed J.C., Takahashi R. (2001). X-linked inhibitor of apoptosis protein (XIAP) inhibits caspase-3 and -7 in distinct modes. J. Biol. Chem..

[24] Eckelman B.P., Salvesen G.S. (2006). The human anti-apoptotic proteins cIAP1 and cIAP2 bind but do not inhibit caspases. J. Biol. Chem..

[25] Eckelman B.P., Salvesen G.S., Scott F.L. (2006). Human inhibitor of apoptosis proteins: Why XIAP is the black sheep of the family. EMBO Rep..

[26] Gyrd-Hansen M., Darding M., Miasari M., Santoro M.M., Zender L., Xue W., Tenev T., da Fonseca P.C., Zvelebil M., Bujnicki J.M. (2008). IAPs contain an evolutionarily conserved ubiquitin-binding domain that regulates NF-kappaB as well as cell survival and oncogenesis. Nat. Cell Biol..

[27] Schile A.J., Garcia-Fernandez M., Steller H. (2008). Regulation of apoptosis by XIAP ubiquitin-ligase activity. Genes Dev..

[28] Rajalingam K., Dikic I. (2009). Inhibitors of apoptosis catch ubiquitin. Biochem. J..

[29] Vucic D., Stennicke H.R., Pisabarro M.T., Salvesen G.S., Dixit V.M. (2000). ML-IAP, a novel inhibitor of apoptosis that is preferentially expressed in human melanomas. Curr. Biol..

[30] Danial N.N., Korsmeyer S.J. (2004). Cell death: Critical control points. Cell.

[31] Lowe S.W., Lin A.W. (2000). Apoptosis in cancer. Carcinogenesis.

[32] Cotter T.G. (2009). Apoptosis and cancer: The genesis of a research field. Nat. Rev. Cancer.

[33] Abbas R., Larisch S. (2020). Targeting XIAP for Promoting Cancer Cell Death-The Story of ARTS and SMAC. Cells.

[34] Bornstein B., Edison N., Gottfried Y., Lev T., Shekhtman A., Gonen H., Rajalingam K., Larisch S. (2012). X-linked Inhibitor of Apoptosis Protein promotes the degradation of its antagonist, the pro-apoptotic ARTS protein. Int. J. Biochem. Cell Biol..

[35] Bornstein B., Gottfried Y., Edison N., Shekhtman A., Lev T., Glaser F., Larisch S. (2011). ARTS binds to a distinct domain in XIAP-BIR3 and promotes apoptosis by a mechanism that is different from other IAP-antagonists. Apoptosis.

[36] Edison N., Curtz Y., Paland N., Mamriev D., Chorubczyk N., Haviv-Reingewertz T., Kfir N., Morgenstern D., Kupervaser M., Kagan J. (2017). Degradation of Bcl-2 by XIAP and ARTS Promotes Apoptosis. Cell Rep..

[37] Gottfried Y., Rotem A., Lotan R., Steller H., Larisch S. (2004). The mitochondrial ARTS protein promotes apoptosis through targeting XIAP. EMBO J..

[38] Lotan R., Rotem A., Gonen H., Finberg J.P., Kemeny S., Steller H., Ciechanover A., Larisch S. (2005). Regulation of the proapoptotic ARTS protein by ubiquitin-mediated degradation. J. Biol. Chem..

[39] Bader M., Steller H. (2009). Regulation of cell death by the ubiquitin-proteasome system. Curr. Opin. Cell Biol..

[40] Vaux D.L., Silke J. (2005). IAPs—The ubiquitin connection. Cell Death Differ..

[41] Vaux D.L., Silke J. (2005). IAPs, RINGs and ubiquitylation. Nat. Rev. Mol. Cell Biol..

[42] Abu Ahmad Y., Oknin-Vaisman A., Bitman-Lotan E., Orian A. (2021). From the Evasion of Degradation to Ubiquitin-Dependent Protein Stabilization. Cells.

[43] Ciechanover A. (2017). Intracellular protein degradation: From a vague idea thru the lysosome and the ubiquitin-proteasome system and onto human diseases and drug targeting. Best Pract. Res. Clin. Haematol..

[44] Collins G.A., Goldberg A.L. (2017). The Logic of the 26S Proteasome. Cell.

[45] Ciechanover A., Orian A., Schwartz A.L. (2000). Ubiquitin-mediated proteolysis: Biological regulation via destruction. Bioessays.

[46] Hershko A., Ciechanover A., Rose I.A. (1979). Resolution of the ATP-dependent proteolytic system from reticulocytes: A component that interacts with ATP. Proc. Natl. Acad. Sci. USA.

[47] Ciechanover A., Heller H., Elias S., Haas A.L., Hershko A. (1980). ATP-dependent conjugation of reticulocyte proteins with the polypeptide required for protein degradation. Proc. Natl. Acad. Sci. USA.

[48] Ciechanover A., Hod Y., Hershko A. (2012). A heat-stable polypeptide component of an ATP-dependent proteolytic system from reticulocytes. 1978. Biochem. Biophys. Res. Commun..

[49] Mayor T., Sharon M., Glickman M.H. (2016). Tuning the proteasome to brighten the end of the journey. Am. J. Physiol Cell Physiol.

[50] Hershko A., Ciechanover A., Heller H., Haas A.L., Rose I.A. (1980). Proposed role of ATP in protein breakdown: Conjugation of protein with multiple chains of the polypeptide of ATP-dependent proteolysis. Proc. Natl. Acad. Sci. USA.

[51] Broemer M., Meier P. (2009). Ubiquitin-mediated regulation of apoptosis. Trends Cell Biol..

[52] Thompson S.J., Loftus L.T., Ashley M.D., Meller R. (2008). Ubiquitin-proteasome system as a modulator of cell fate. Curr. Opin. Pharmacol..

[53] Argentini M., Barboule N., Wasylyk B. (2000). The contribution of the RING finger domain of MDM2 to cell cycle progression. Oncogene.

[54] Huang H., Joazeiro C.A., Bonfoco E., Kamada S., Leverson J.D., Hunter T. (2000). The inhibitor of apoptosis, cIAP2, functions as a ubiquitin-protein ligase and promotes in vitro monoubiquitination of caspases 3 and 7. J. Biol. Chem..

[55] Suzuki Y., Nakabayashi Y., Takahashi R. (2001). Ubiquitin-protein ligase activity of X-linked inhibitor of apoptosis protein promotes proteasomal degradation of caspase-3 and enhances its anti-apoptotic effect in Fas-induced cell death. Proc. Natl. Acad. Sci. USA.

[56] Wilson R., Goyal L., Ditzel M., Zachariou A., Baker D.A., Agapite J., Steller H., Meier P. (2002). The DIAP1 RING finger mediates ubiquitination of Dronc and is indispensable for regulating apoptosis. Nat. Cell Biol..

[57] Haglund K., Dikic I. (2005). Ubiquitylation and cell signaling. EMBO J..

[58] Nakayama K.I., Nakayama K. (2006). Ubiquitin ligases: Cell-cycle control and cancer. Nat. Rev. Cancer.

[59] Mukhopadhyay D., Riezman H. (2007). Proteasome-independent functions of ubiquitin in endocytosis and signaling. Science.

[60] Ikeda F., Dikic I. (2008). Atypical ubiquitin chains: New molecular signals. ‘Protein Modifications: Beyond the Usual Suspects’ review series. EMBO Rep..

[61] Swatek K.N., Komander D. (2016). Ubiquitin modifications. Cell Res..

[62] Miranda M., Sorkin A. (2007). Regulation of receptors and transporters by ubiquitination: New insights into surprisingly similar mechanisms. Mol. Interv..

[63] Livneh I., Kravtsova-Ivantsiv Y., Braten O., Kwon Y.T., Ciechanover A. (2017). Monoubiquitination joins polyubiquitination as an esteemed proteasomal targeting signal. Bioessays.

[64] Nakagawa T., Nakayama K. (2015). Protein monoubiquitylation: Targets and diverse functions. Genes Cells.

[65] Shabek N., Herman-Bachinsky Y., Buchsbaum S., Lewinson O., Haj-Yahya M., Hejjaoui M., Lashuel H.A., Sommer T., Brik A., Ciechanover A. (2012). The size of the proteasomal substrate determines whether its degradation will be mediated by mono- or polyubiquitylation. Mol. Cell.

[66] Shabek N., Herman-Bachinsky Y., Ciechanover A. (2009). Ubiquitin degradation with its substrate, or as a monomer in a ubiquitination-independent mode, provides clues to proteasome regulation. Proc. Natl. Acad. Sci. USA.

[67] Shabek N., Iwai K., Ciechanover A. (2007). Ubiquitin is degraded by the ubiquitin system as a monomer and as part of its conjugated target. Biochem. Biophys. Res. Commun..

[68] Ohtake F., Saeki Y., Ishido S., Kanno J., Tanaka K. (2016). The K48-K63 Branched Ubiquitin Chain Regulates NF-kappaB Signaling. Mol. Cell.

[69] Yau R., Rape M. (2016). The increasing complexity of the ubiquitin code. Nat. Cell Biol..

[70] Jacobson A.D., Zhang N.Y., Xu P., Han K.J., Noone S., Peng J., Liu C.W. (2009). The lysine 48 and lysine 63 ubiquitin conjugates are processed differently by the 26 s proteasome. J. Biol. Chem..

[71] Kim W., Bennett E.J., Huttlin E.L., Guo A., Li J., Possemato A., Sowa M.E., Rad R., Rush J., Comb M.J. (2011). Systematic and quantitative assessment of the ubiquitin-modified proteome. Mol. Cell.

[72] Xu P., Duong D.M., Seyfried N.T., Cheng D., Xie Y., Robert J., Rush J., Hochstrasser M., Finley D., Peng J. (2009). Quantitative proteomics reveals the function of unconventional ubiquitin chains in proteasomal degradation. Cell.

[73] Gendron J.M., Webb K., Yang B., Rising L., Zuzow N., Bennett E.J. (2016). Using the Ubiquitin-modified Proteome to Monitor Distinct and Spatially Restricted Protein Homeostasis Dysfunction. Mol. Cell Proteom..

[74] Nathan J.A., Kim H.T., Ting L., Gygi S.P., Goldberg A.L. (2013). Why do cellular proteins linked to K63-polyubiquitin chains not associate with proteasomes?. EMBO J..

[75] Ashwell J.D. (2008). TWEAKing death. J. Cell Biol..

[76] Komander D., Rape M. (2012). The ubiquitin code. Annu. Rev. Biochem..

[77] Husnjak K., Dikic I. (2012). Ubiquitin-binding proteins: Decoders of ubiquitin-mediated cellular functions. Annu. Rev. Biochem..

[78] Wolf D.H., Hilt W. (2004). The proteasome: A proteolytic nanomachine of cell regulation and waste disposal. Biochim. Biophys. Acta.

[79] Pickart C.M. (2001). Mechanisms underlying ubiquitination. Annu. Rev. Biochem..

[80] Kleiger G., Mayor T. (2014). Perilous journey: A tour of the ubiquitin-proteasome system. Trends Cell Biol..

[81] Zheng N., Shabek N. (2017). Ubiquitin Ligases: Structure, Function, and Regulation. Annu. Rev. Biochem..

[82] David Y., Ternette N., Edelmann M.J., Ziv T., Gayer B., Sertchook R., Dadon Y., Kessler B.M., Navon A. (2011). E3 ligases determine ubiquitination site and conjugate type by enforcing specificity on E2 enzymes. J. Biol. Chem..

[83] Ardley H.C., Robinson P.A. (2005). E3 ubiquitin ligases. Essays Biochem..

[84] Berndsen C.E., Wolberger C. (2014). New insights into ubiquitin E3 ligase mechanism. Nat. Struct. Mol. Biol..

[85] Morreale F.E., Walden H. (2016). Types of Ubiquitin Ligases. Cell.

[86] Sluimer J., Distel B. (2018). Regulating the human HECT E3 ligases. Cell Mol. Life Sci..

[87] Weber J., Polo S., Maspero E. (2019). HECT E3 Ligases: A Tale With Multiple Facets. Front. Physiol..

[88] de Wilt L.H., Jansen G., Assaraf Y.G., van Meerloo J., Cloos J., Schimmer A.D., Chan E.T., Kirk C.J., Peters G.J., Kruyt F.A. (2012). Proteasome-based mechanisms of intrinsic and acquired bortezomib resistance in non-small cell lung cancer. Biochem. Pharmacol..

[89] Franke N.E., Kaspers G.L., Assaraf Y.G., van Meerloo J., Niewerth D., Kessler F.L., Poddighe P.J., Kole J., Smeets S.J., Ylstra B. (2016). Exocytosis of polyubiquitinated proteins in bortezomib-resistant leukemia cells: A role for MARCKS in acquired resistance to proteasome inhibitors. Oncotarget.

[90] Franke N.E., Niewerth D., Assaraf Y.G., van Meerloo J., Vojtekova K., van Zantwijk C.H., Zweegman S., Chan E.T., Kirk C.J., Geerke D.P. (2012). Impaired bortezomib binding to mutant beta5 subunit of the proteasome is the underlying basis for bortezomib resistance in leukemia cells. Leukemia.

[91] Jeon Y.K., Kim C.K., Koh J., Chung D.H., Ha G.H. (2016). Pellino-1 confers chemoresistance in lung cancer cells by upregulating cIAP2 through Lys63-mediated polyubiquitination. Oncotarget.

[92] Nelson J.K., Cook E.C., Loregger A., Hoeksema M.A., Scheij S., Kovacevic I., Hordijk P.L., Ovaa H., Zelcer N. (2016). Deubiquitylase Inhibition Reveals Liver X Receptor-independent Transcriptional Regulation of the E3 Ubiquitin Ligase IDOL and Lipoprotein Uptake. J. Biol. Chem..

[93] Niewerth D., Jansen G., Assaraf Y.G., Zweegman S., Kaspers G.J., Cloos J. (2015). Molecular basis of resistance to proteasome inhibitors in hematological malignancies. Drug Resist. Updates.

[94] Niewerth D., Jansen G., Riethoff L.F., van Meerloo J., Kale A.J., Moore B.S., Assaraf Y.G., Anderl J.L., Zweegman S., Kaspers G.J. (2014). Antileukemic activity and mechanism of drug resistance to the marine Salinispora tropica proteasome inhibitor salinosporamide A (Marizomib). Mol. Pharmacol..

[95] Niewerth D., van Meerloo J., Jansen G., Assaraf Y.G., Hendrickx T.C., Kirk C.J., Anderl J.L., Zweegman S., Kaspers G.J., Cloos J. (2014). Anti-leukemic activity and mechanisms underlying resistance to the novel immunoproteasome inhibitor PR-924. Biochem. Pharmacol..

[96] Oerlemans R., Franke N.E., Assaraf Y.G., Cloos J., van Zantwijk I., Berkers C.R., Scheffer G.L., Debipersad K., Vojtekova K., Lemos C. (2008). Molecular basis of bortezomib resistance: Proteasome subunit beta5 (PSMB5) gene mutation and overexpression of PSMB5 protein. Blood.

[97] Petzold G., Fischer E.S., Thoma N.H. (2016). Structural basis of lenalidomide-induced CK1alpha degradation by the CRL4(CRBN) ubiquitin ligase. Nature.

[98] Tanaka N., Kosaka T., Miyazaki Y., Mikami S., Niwa N., Otsuka Y., Minamishima Y.A., Mizuno R., Kikuchi E., Miyajima A. (2016). Acquired platinum resistance involves epithelial to mesenchymal transition through ubiquitin ligase FBXO32 dysregulation. JCI Insight.

[99] Xu Q., Hou Y.X., Langlais P., Erickson P., Zhu J., Shi C.X., Luo M., Zhu Y., Xu Y., Mandarino L.J. (2016). Expression of the cereblon binding protein argonaute 2 plays an important role for multiple myeloma cell growth and survival. BMC Cancer.

[100] Yoshino S., Hara T., Nakaoka H.J., Kanamori A., Murakami Y., Seiki M., Sakamoto T. (2016). The ERK signaling target RNF126 regulates anoikis resistance in cancer cells by changing the mitochondrial metabolic flux. Cell Discov..

[101] Zhang X.D., Baladandayuthapani V., Lin H., Mulligan G., Li B., Esseltine D.W., Qi L., Xu J., Hunziker W., Barlogie B. (2016). Tight Junction Protein 1 Modulates Proteasome Capacity and Proteasome Inhibitor Sensitivity in Multiple Myeloma via EGFR/JAK1/STAT3 Signaling. Cancer Cell.

[102] Cao B., Mao X. (2011). The ubiquitin-proteasomal system is critical for multiple myeloma: Implications in drug discovery. Am. J. Blood Res..

[103] Gandhi A.K., Kang J., Havens C.G., Conklin T., Ning Y., Wu L., Ito T., Ando H., Waldman M.F., Thakurta A. (2014). Immunomodulatory agents lenalidomide and pomalidomide co-stimulate T cells by inducing degradation of T cell repressors Ikaros and Aiolos via modulation of the E3 ubiquitin ligase complex CRL4(CRBN.). Br. J. Haematol..

[104] Huang H., Weng H., Dong B., Zhao P., Zhou H., Qu L. (2017). Oridonin Triggers Chaperon-mediated Proteasomal Degradation of BCR-ABL in Leukemia. Sci. Rep..

[105] Lu G., Middleton R.E., Sun H., Naniong M., Ott C.J., Mitsiades C.S., Wong K.K., Bradner J.E., Kaelin W.G. (2014). The myeloma drug lenalidomide promotes the cereblon-dependent destruction of Ikaros proteins. Science.

[106] Micel L.N., Tentler J.J., Smith P.G., Eckhardt G.S. (2013). Role of ubiquitin ligases and the proteasome in oncogenesis: Novel targets for anticancer therapies. J. Clin. Oncol..

[107] Wu B., Chu X., Feng C., Hou J., Fan H., Liu N., Li C., Kong X., Ye X., Meng S. (2015). Heat shock protein gp96 decreases p53 stability by regulating Mdm2 E3 ligase activity in liver cancer. Cancer Lett..

[108] Yerlikaya A., Yontem M. (2013). The significance of ubiquitin proteasome pathway in cancer development. Recent Pat. Anticancer Drug Discov..

[109] Lipkowitz S., Weissman A.M. (2011). RINGs of good and evil: RING finger ubiquitin ligases at the crossroads of tumour suppression and oncogenesis. Nat. Rev. Cancer.

[110] Ohi M.D., Vander Kooi C.W., Rosenberg J.A., Chazin W.J., Gould K.L. (2003). Structural insights into the U-box, a domain associated with multi-ubiquitination. Nat. Struct. Biol..

[111] Metzger M.B., Pruneda J.N., Klevit R.E., Weissman A.M. (2014). RING-type E3 ligases: Master manipulators of E2 ubiquitin-conjugating enzymes and ubiquitination. Biochim. Biophys. Acta.

[112] Eldridge A.G., O’Brien T. (2010). Therapeutic strategies within the ubiquitin proteasome system. Cell Death Differ..

[113] Sarikas A., Hartmann T., Pan Z.Q. (2011). The cullin protein family. Genome Biol..

[114] Spratt D.E., Walden H., Shaw G.S. (2014). RBR E3 ubiquitin ligases: New structures, new insights, new questions. Biochem. J..

[115] Walden H., Rittinger K. (2018). RBR ligase-mediated ubiquitin transfer: A tale with many twists and turns. Nat. Struct. Mol. Biol..

[116] Vasudevan D., Ryoo H.D. (2015). Regulation of Cell Death by IAPs and Their Antagonists. Curr. Top. Dev. Biol..

[117] Bergmann A. (2010). The role of ubiquitylation for the control of cell death in Drosophila. Cell Death Differ..

[118] Schwartz L.M., Myer A., Kosz L., Engelstein M., Maier C. (1990). Activation of polyubiquitin gene expression during developmentally programmed cell death. Neuron.

[119] Xu T., Jiang X., Denton D., Kumar S. (2020). Ecdysone controlled cell and tissue deletion. Cell Death Differ..

[120] Lockshin R.A., Williams C.M. (1965). Programmed Cell Death--I. Cytology of Degeneration in the Intersegmental Muscles of the Pernyi Silkmoth. J. Insect Physiol..

[121] Kaelin W.G. (1999). The p53 gene family. Oncogene.

[122] Oren M. (1999). Regulation of the p53 tumor suppressor protein. J. Biol. Chem..

[123] Labrecque S., Naor N., Thomson D., Matlashewski G. (1993). Analysis of the Anti-p53 Antibody Response in Cancer Patients. Cancer Res..

[124] Tokino T., Nakamura Y. (2000). The role of p53-target genes in human cancer. Crit. Rev. Oncol. Hematol.

[125] Yang L., Song T., Cheng Q., Chen L., Chen J. (2019). Mutant p53 Sequestration of the MDM2 Acidic Domain Inhibits E3 Ligase Activity. Mol. Cell Biol..

[126] Khoo K.H., Verma C.S., Lane D.P. (2014). Drugging the p53 pathway: Understanding the route to clinical efficacy. Nat. Rev. Drug Discov..

[127] Powell E., Piwnica-Worms D., Piwnica-Worms H. (2014). Contribution of p53 to metastasis. Cancer Discov..

[128] Joerger A.C., Fersht A.R. (2016). The p53 Pathway: Origins, Inactivation in Cancer, and Emerging Therapeutic Approaches. Annu. Rev. Biochem..

[129] Klimovich B., Mutlu S., Schneikert J., Elmshauser S., Klimovich M., Nist A., Mernberger M., Timofeev O., Stiewe T. (2019). Loss of p53 function at late stages of tumorigenesis confers ARF-dependent vulnerability to p53 reactivation therapy. Proc. Natl. Acad. Sci. USA.

[130] Brooks C.L., Gu W. (2011). p53 regulation by ubiquitin. FEBS Lett..

[131] Hock A.K., Vousden K.H. (2014). The role of ubiquitin modification in the regulation of p53. Biochim. Biophys. Acta.

[132] Levine A.J., Oren M. (2009). The first 30 years of p53: Growing ever more complex. Nat. Rev. Cancer.

[133] Aylon Y., Oren M. (2007). Living with p53, dying of p53. Cell.

[134] Baresova P., Musilova J., Pitha P.M., Lubyova B. (2014). p53 tumor suppressor protein stability and transcriptional activity are targeted by Kaposi’s sarcoma-associated herpesvirus-encoded viral interferon regulatory factor 3. Mol. Cell Biol..

[135] Giaccia A.J., Kastan M.B. (1998). The complexity of p53 modulation: Emerging patterns from divergent signals. Genes Dev..

[136] Maki C.G., Howley P.M. (1997). Ubiquitination of p53 and p21 is differentially affected by ionizing and UV radiation. Mol. Cell Biol..

[137] Price B.D., Calderwood S.K. (1993). Increased sequence-specific p53-DNA binding activity after DNA damage is attenuated by phorbol esters. Oncogene.

[138] Maltzman W., Czyzyk L. (1984). UV irradiation stimulates levels of p53 cellular tumor antigen in nontransformed mouse cells. Mol. Cell Biol..

[139] Vogelstein B., Lane D., Levine A.J. (2000). Surfing the p53 network. Nature.

[140] Wang X., Simpson E.R., Brown K.A. (2015). p53: Protection against Tumor Growth beyond Effects on Cell Cycle and Apoptosis. Cancer Res..

[141] Haupt Y., Maya R., Kazaz A., Oren M. (1997). Mdm2 promotes the rapid degradation of p53. Nature.

[142] Kubbutat M.H., Jones S.N., Vousden K.H. (1997). Regulation of p53 stability by Mdm2. Nature.

[143] Tao W., Levine A.J. (1999). Nucleocytoplasmic shuttling of oncoprotein Hdm2 is required for Hdm2-mediated degradation of p53. Proc. Natl. Acad. Sci. USA.

[144] Nag S., Zhang X., Srivenugopal K.S., Wang M.H., Wang W., Zhang R. (2014). Targeting MDM2-p53 interaction for cancer therapy: Are we there yet?. Curr. Med. Chem..

[145] Poyurovsky M.V., Katz C., Laptenko O., Beckerman R., Lokshin M., Ahn J., Byeon I.J., Gabizon R., Mattia M., Zupnick A. (2010). The C terminus of p53 binds the N-terminal domain of MDM2. Nat. Struct. Mol. Biol..

[146] Chi S.W., Lee S.H., Kim D.H., Ahn M.J., Kim J.S., Woo J.Y., Torizawa T., Kainosho M., Han K.H. (2005). Structural details on mdm2-p53 interaction. J. Biol. Chem..

[147] Jones S.N., Roe A.E., Donehower L.A., Bradley A. (1995). Rescue of embryonic lethality in Mdm2-deficient mice by absence of p53. Nature.

[148] Montes de Oca Luna R., Wagner D.S., Lozano G. (1995). Rescue of early embryonic lethality in mdm2-deficient mice by deletion of p53. Nature.

[149] Barak Y., Juven T., Haffner R., Oren M. (1993). mdm2 expression is induced by wild type p53 activity. EMBO J..

[150] Perry M.E., Piette J., Zawadzki J.A., Harvey D., Levine A.J. (1993). The mdm-2 gene is induced in response to UV light in a p53-dependent manner. Proc. Natl. Acad. Sci. USA.

[151] Wu X., Bayle J.H., Olson D., Levine A.J. (1993). The p53-mdm-2 autoregulatory feedback loop. Genes Dev..

[152] Fang S., Jensen J.P., Ludwig R.L., Vousden K.H., Weissman A.M. (2000). Mdm2 is a RING finger-dependent ubiquitin protein ligase for itself and p53. J. Biol. Chem..

[153] Honda R., Yasuda H. (2000). Activity of MDM2, a ubiquitin ligase, toward p53 or itself is dependent on the RING finger domain of the ligase. Oncogene.

[154] Poyurovsky M.V., Priest C., Kentsis A., Borden K.L., Pan Z.Q., Pavletich N., Prives C. (2007). The Mdm2 RING domain C-terminus is required for supramolecular assembly and ubiquitin ligase activity. EMBO J..

[155] Linke K., Mace P.D., Smith C.A., Vaux D.L., Silke J., Day C.L. (2008). Structure of the MDM2/MDMX RING domain heterodimer reveals dimerization is required for their ubiquitylation in trans. Cell Death Differ..

[156] Sharp D.A., Kratowicz S.A., Sank M.J., George D.L. (1999). Stabilization of the MDM2 oncoprotein by interaction with the structurally related MDMX protein. J. Biol. Chem..

[157] Liu T., Zhang H., Xiong J., Yi S., Gu L., Zhou M. (2015). Inhibition of MDM2 homodimerization by XIAP IRES stabilizes MDM2, influencing cancer cell survival. Mol. Cancer.

[158] Stad R., Little N.A., Xirodimas D.P., Frenk R., van der Eb A.J., Lane D.P., Saville M.K., Jochemsen A.G. (2001). Mdmx stabilizes p53 and Mdm2 via two distinct mechanisms. EMBO Rep..

[159] de Polo A., Luo Z., Gerarduzzi C., Chen X., Little J.B., Yuan Z.M. (2017). AXL receptor signalling suppresses p53 in melanoma through stabilization of the MDMX-MDM2 complex. J. Mol. Cell Biol..

[160] Shvarts A., Steegenga W.T., Riteco N., van Laar T., Dekker P., Bazuine M., van Ham R.C., van der Houven van Oordt W., Hateboer G., van der Eb A.J. (1996). MDMX: A novel p53-binding protein with some functional properties of MDM2. EMBO J..

[161] Singh R.K., Iyappan S., Scheffner M. (2007). Hetero-oligomerization with MdmX rescues the ubiquitin/Nedd8 ligase activity of RING finger mutants of Mdm2. J. Biol. Chem..

[162] Finch R.A., Donoviel D.B., Potter D., Shi M., Fan A., Freed D.D., Wang C.Y., Zambrowicz B.P., Ramirez-Solis R., Sands A.T. (2002). mdmx is a negative regulator of p53 activity in vivo. Cancer Res..

[163] Parant J., Chavez-Reyes A., Little N.A., Yan W., Reinke V., Jochemsen A.G., Lozano G. (2001). Rescue of embryonic lethality in Mdm4-null mice by loss of Trp53 suggests a nonoverlapping pathway with MDM2 to regulate p53. Nat. Genet..

[164] Migliorini D., Lazzerini Denchi E., Danovi D., Jochemsen A., Capillo M., Gobbi A., Helin K., Pelicci P.G., Marine J.C. (2002). Mdm4 (Mdmx) regulates p53-induced growth arrest and neuronal cell death during early embryonic mouse development. Mol. Cell Biol..

[165] Gu L., Zhang H., Liu T., Zhou S., Du Y., Xiong J., Yi S., Qu C.K., Fu H., Zhou M. (2016). Discovery of Dual Inhibitors of MDM2 and XIAP for Cancer Treatment. Cancer Cell.

[166] Zhang X.W., Wang X.F., Ni S.J., Qin W., Zhao L.Q., Hua R.X., Lu Y.W., Li J., Dimri G.P., Guo W.J. (2015). UBTD1 induces cellular senescence through an UBTD1-Mdm2/p53 positive feedback loop. J. Pathol..

[167] Leng R.P., Lin Y., Ma W., Wu H., Lemmers B., Chung S., Parant J.M., Lozano G., Hakem R., Benchimol S. (2003). Pirh2, a p53-induced ubiquitin-protein ligase, promotes p53 degradation. Cell.

[168] Daks A., Petukhov A., Fedorova O., Shuvalov O., Merkulov V., Vasileva E., Antonov A., Barlev N.A. (2016). E3 ubiquitin ligase Pirh2 enhances tumorigenic properties of human non-small cell lung carcinoma cells. Genes Cancer.

[169] Hakem A., Bohgaki M., Lemmers B., Tai E., Salmena L., Matysiak-Zablocki E., Jung Y.S., Karaskova J., Kaustov L., Duan S. (2011). Role of Pirh2 in mediating the regulation of p53 and c-Myc. PLoS Genet..

[170] Bao Y., Wu X., Yuan D., Shi W., Shi J. (2017). High Expression of Pirh2 is Associated with Poor Prognosis in Glioma. Cell Mol. NeuroBiol..

[171] Yang S., Chen Y., Sun F., Ni Q., Wang H., Huang Y., Zhang C., Liu K., Wang S., Qiu J. (2016). Downregulated PIRH2 Can Decrease the Proliferation of Breast Cancer Cells. Arch. Med. Res..

[172] Hu L., Liu M., Chen L., Chan T.H., Wang J., Huo K.K., Zheng B.J., Xie D., Guan X.Y. (2012). SCYL1 binding protein 1 promotes the ubiquitin-dependent degradation of Pirh2 and has tumor-suppressive function in the development of hepatocellular carcinoma. Carcinogenesis.

[173] Dornan D., Wertz I., Shimizu H., Arnott D., Frantz G.D., Dowd P., O’Rourke K., Koeppen H., Dixit V.M. (2004). The ubiquitin ligase COP1 is a critical negative regulator of p53. Nature.

[174] Migliorini D., Bogaerts S., Defever D., Vyas R., Denecker G., Radaelli E., Zwolinska A., Depaepe V., Hochepied T., Skarnes W.C. (2011). Cop1 constitutively regulates c-Jun protein stability and functions as a tumor suppressor in mice. J. Clin. Investig..

[175] Chen D., Kon N., Li M., Zhang W., Qin J., Gu W. (2005). ARF-BP1/Mule is a critical mediator of the ARF tumor suppressor. Cell.

[176] Cambiaghi V., Giuliani V., Lombardi S., Marinelli C., Toffalorio F., Pelicci P.G. (2012). TRIM proteins in cancer. Adv. Exp. Med. Biol..

[177] Wang C., Ivanov A., Chen L., Fredericks W.J., Seto E., Rauscher F.J., Chen J. (2005). MDM2 interaction with nuclear corepressor KAP1 contributes to p53 inactivation. EMBO J..

[178] Okamoto K., Kitabayashi I., Taya Y. (2006). KAP1 dictates p53 response induced by chemotherapeutic agents via Mdm2 interaction. Biochem. Biophys. Res. Commun..

[179] Jiang J., Ballinger C.A., Wu Y., Dai Q., Cyr D.M., Hohfeld J., Patterson C. (2001). CHIP is a U-box-dependent E3 ubiquitin ligase: Identification of Hsc70 as a target for ubiquitylation. J. Biol. Chem..

[180] Xu Z., Devlin K.I., Ford M.G., Nix J.C., Qin J., Misra S. (2006). Structure and interactions of the helical and U-box domains of CHIP, the C terminus of HSP70 interacting protein. Biochemistry.

[181] Maan M., Pati U. (2018). CHIP promotes autophagy-mediated degradation of aggregating mutant p53 in hypoxic conditions. FEBS J..

[182] Esser C., Scheffner M., Hohfeld J. (2005). The chaperone-associated ubiquitin ligase CHIP is able to target p53 for proteasomal degradation. J. Biol. Chem..

[183] Jain A.K., Barton M.C. (2010). Making sense of ubiquitin ligases that regulate p53. Cancer Biol. Ther..

[184] O’Connor L., Harris A.W., Strasser A. (2000). CD95 (Fas/APO-1) and p53 signal apoptosis independently in diverse cell types. Cancer Res..

[185] Liu X., Yue P., Khuri F.R., Sun S.Y. (2004). p53 upregulates death receptor 4 expression through an intronic p53 binding site. Cancer Res..

[186] Oda E., Ohki R., Murasawa H., Nemoto J., Shibue T., Yamashita T., Tokino T., Taniguchi T., Tanaka N. (2000). Noxa, a BH3-only member of the Bcl-2 family and candidate mediator of p53-induced apoptosis. Science.

[187] Nakano K., Vousden K.H. (2001). PUMA, a novel proapoptotic gene, is induced by p53. Mol. Cell.

[188] Fridman J.S., Lowe S.W. (2003). Control of apoptosis by p53. Oncogene.

[189] Meulmeester E., Jochemsen A.G. (2008). p53: A guide to apoptosis. Curr. Cancer Drug Targets.

[190] Hao Q., Chen J., Liao J., Huang Y., Larisch S., Zeng S.X., Lu H., Zhou X. (2020). p53 induces ARTS to promote mitochondrial apoptosis. bioRxiv.

[191] Tait S.W., Green D.R. (2010). Mitochondria and cell death: Outer membrane permeabilization and beyond. Nat. Rev. Mol. Cell Biol..

[192] Chen J. (2016). The Cell-Cycle Arrest and Apoptotic Functions of p53 in Tumor Initiation and Progression. Cold Spring Harb. Perspect. Med..

[193] Edison N., Zuri D., Maniv I., Bornstein B., Lev T., Gottfried Y., Kemeny S., Garcia-Fernandez M., Kagan J., Larisch S. (2012). The IAP-antagonist ARTS initiates caspase activation upstream of cytochrome C and SMAC/Diablo. Cell Death Differ..

[194] Sun L., Shi L., Li W., Yu W., Liang J., Zhang H., Yang X., Wang Y., Li R., Yao X. (2009). JFK, a Kelch domain-containing F-box protein, links the SCF complex to p53 regulation. Proc. Natl. Acad. Sci. USA.

[195] Nag A., Bagchi S., Raychaudhuri P. (2004). Cul4A physically associates with MDM2 and participates in the proteolysis of p53. Cancer Res..

[196] Kopanja D., Stoyanova T., Okur M.N., Huang E., Bagchi S., Raychaudhuri P. (2009). Proliferation defects and genome instability in cells lacking Cul4A. Oncogene.

[197] Banks D., Wu M., Higa L.A., Gavrilova N., Quan J., Ye T., Kobayashi R., Sun H., Zhang H. (2006). L2DTL/CDT2 and PCNA interact with p53 and regulate p53 polyubiquitination and protein stability through MDM2 and CUL4A/DDB1 complexes. Cell Cycle.

[198] Querido E., Blanchette P., Yan Q., Kamura T., Morrison M., Boivin D., Kaelin W.G., Conaway R.C., Conaway J.W., Branton P.E. (2001). Degradation of p53 by adenovirus E4orf6 and E1B55K proteins occurs via a novel mechanism involving a Cullin-containing complex. Genes Dev..

[199] Yamasaki S., Yagishita N., Sasaki T., Nakazawa M., Kato Y., Yamadera T., Bae E., Toriyama S., Ikeda R., Zhang L. (2007). Cytoplasmic destruction of p53 by the endoplasmic reticulum-resident ubiquitin ligase ‘Synoviolin’. EMBO J..

[200] Rajendra R., Malegaonkar D., Pungaliya P., Marshall H., Rasheed Z., Brownell J., Liu L.F., Lutzker S., Saleem A., Rubin E.H. (2004). Topors functions as an E3 ubiquitin ligase with specific E2 enzymes and ubiquitinates p53. J. Biol. Chem..

[201] Allton K., Jain A.K., Herz H.M., Tsai W.W., Jung S.Y., Qin J., Bergmann A., Johnson R.L., Barton M.C. (2009). Trim24 targets endogenous p53 for degradation. Proc. Natl. Acad. Sci. USA.

[202] Doyle J.M., Gao J., Wang J., Yang M., Potts P.R. (2010). MAGE-RING protein complexes comprise a family of E3 ubiquitin ligases. Mol. Cell.

[203] Zhang L., Huang N.J., Chen C., Tang W., Kornbluth S. (2012). Ubiquitylation of p53 by the APC/C inhibitor Trim39. Proc. Natl. Acad. Sci. USA.

[204] Li Y., Ma C., Zhou T., Liu Y., Sun L., Yu Z. (2016). TRIM65 negatively regulates p53 through ubiquitination. Biochem. Biophys. Res. Commun..

[205] Yang W., Rozan L.M., McDonald E.R., Navaraj A., Liu J.J., Matthew E.M., Wang W., Dicker D.T., El-Deiry W.S. (2007). CARPs are ubiquitin ligases that promote MDM2-independent p53 and phospho-p53ser20 degradation. J. Biol. Chem..

[206] Zhang X., Li C.F., Zhang L., Wu C.Y., Han L., Jin G., Rezaeian A.H., Han F., Liu C., Xu C. (2016). TRAF6 Restricts p53 Mitochondrial Translocation, Apoptosis, and Tumor Suppression. Mol. Cell.

[207] Wang L., Wang L., Zhang S., Qu G., Zhang D., Li S., Liu S. (2013). Downregulation of ubiquitin E3 ligase TNF receptor-associated factor 7 leads to stabilization of p53 in breast cancer. Oncol. Rep..

[208] Su W.J., Fang J.S., Cheng F., Liu C., Zhou F., Zhang J. (2013). RNF2/Ring1b negatively regulates p53 expression in selective cancer cell types to promote tumor development. Proc. Natl. Acad. Sci. USA.

[209] Shen J., Li P., Shao X., Yang Y., Liu X., Feng M., Yu Q., Hu R., Wang Z. (2018). The E3 Ligase RING1 Targets p53 for Degradation and Promotes Cancer Cell Proliferation and Survival. Cancer Res..

[210] Cory S., Huang D.C., Adams J.M. (2003). The Bcl-2 family: Roles in cell survival and oncogenesis. Oncogene.

[211] Gross A., McDonnell J.M., Korsmeyer S.J. (1999). BCL-2 family members and the mitochondria in apoptosis. Genes Dev..

[212] Adams J.M., Cory S. (1998). The Bcl-2 protein family: Arbiters of cell survival. Science.

[213] Youle R.J., Strasser A. (2008). The BCL-2 protein family: Opposing activities that mediate cell death. Nat. Rev. Mol. Cell Biol..

[214] Siddiqui W.A., Ahad A., Ahsan H. (2015). The mystery of BCL2 family: Bcl-2 proteins and apoptosis: An update. Arch. Toxicol..

[215] Vaux D.L., Cory S., Adams J.M. (1988). Bcl-2 gene promotes haemopoietic cell survival and cooperates with c-myc to immortalize pre-B cells. Nature.

[216] Castle V.P., Heidelberger K.P., Bromberg J., Ou X., Dole M., Nunez G. (1993). Expression of the apoptosis-suppressing protein bcl-2, in neuroblastoma is associated with unfavorable histology and N-myc amplification. Am. J. Pathol..

[217] Krajewska M., Krajewski S., Epstein J.I., Shabaik A., Sauvageot J., Song K., Kitada S., Reed J.C. (1996). Immunohistochemical analysis of bcl-2, bax, bcl-X, and mcl-1 expression in prostate cancers. Am. J. Pathol..

[218] Robertson L.E., Plunkett W., McConnell K., Keating M.J., McDonnell T.J. (1996). Bcl-2 expression in chronic lymphocytic leukemia and its correlation with the induction of apoptosis and clinical outcome. Leukemia.

[219] Dimmeler S., Breitschopf K., Haendeler J., Zeiher A.M. (1999). Dephosphorylation targets Bcl-2 for ubiquitin-dependent degradation: A link between the apoptosome and the proteasome pathway. J. Exp. Med..

[220] Kassi E., Sourlingas T.G., Spiliotaki M., Papoutsi Z., Pratsinis H., Aligiannis N., Moutsatsou P. (2009). Ursolic acid triggers apoptosis and Bcl-2 downregulation in MCF-7 breast cancer cells. Cancer Investig..

[221] Wang L., Chanvorachote P., Toledo D., Stehlik C., Mercer R.R., Castranova V., Rojanasakul Y. (2008). Peroxide is a key mediator of Bcl-2 down-regulation and apoptosis induction by cisplatin in human lung cancer cells. Mol. Pharmacol..

[222] Zhong Q., Gao W., Du F., Wang X. (2005). Mule/ARF-BP1, a BH3-only E3 ubiquitin ligase, catalyzes the polyubiquitination of Mcl-1 and regulates apoptosis. Cell.

[223] Mojsa B., Lassot I., Desagher S. (2014). Mcl-1 ubiquitination: Unique regulation of an essential survival protein. Cells.

[224] Willis S.N., Chen L., Dewson G., Wei A., Naik E., Fletcher J.I., Adams J.M., Huang D.C. (2005). Proapoptotic Bak is sequestered by Mcl-1 and Bcl-xL, but not Bcl-2, until displaced by BH3-only proteins. Genes Dev..

[225] Warr M.R., Acoca S., Liu Z., Germain M., Watson M., Blanchette M., Wing S.S., Shore G.C. (2005). BH3-ligand regulates access of MCL-1 to its E3 ligase. FEBS Lett..

[226] Magiera M.M., Mora S., Mojsa B., Robbins I., Lassot I., Desagher S. (2013). Trim17-mediated ubiquitination and degradation of Mcl-1 initiate apoptosis in neurons. Cell Death Differ..

[227] Carroll R.G., Hollville E., Martin S.J. (2014). Parkin sensitizes toward apoptosis induced by mitochondrial depolarization through promoting degradation of Mcl-1. Cell Rep..

[228] Inuzuka H., Shaik S., Onoyama I., Gao D., Tseng A., Maser R.S., Zhai B., Wan L., Gutierrez A., Lau A.W. (2011). SCF(FBW7) regulates cellular apoptosis by targeting MCL1 for ubiquitylation and destruction. Nature.

[229] Wertz I.E., Kusam S., Lam C., Okamoto T., Sandoval W., Anderson D.J., Helgason E., Ernst J.A., Eby M., Liu J. (2011). Sensitivity to antitubulin chemotherapeutics is regulated by MCL1 and FBW7. Nature.

[230] Ding Q., He X., Hsu J.M., Xia W., Chen C.T., Li L.Y., Lee D.F., Liu J.C., Zhong Q., Wang X. (2007). Degradation of Mcl-1 by beta-TrCP mediates glycogen synthase kinase 3-induced tumor suppression and chemosensitization. Mol. Cell Biol..

[231] Allan L.A., Skowyra A., Rogers K.I., Zeller D., Clarke P.R. (2018). Atypical APC/C-dependent degradation of Mcl-1 provides an apoptotic timer during mitotic arrest. EMBO J..

[232] Harley M.E., Allan L.A., Sanderson H.S., Clarke P.R. (2010). Phosphorylation of Mcl-1 by CDK1-cyclin B1 initiates its Cdc20-dependent destruction during mitotic arrest. EMBO J..

[233] Azakir B.A., Desrochers G., Angers A. (2010). The ubiquitin ligase Itch mediates the antiapoptotic activity of epidermal growth factor by promoting the ubiquitylation and degradation of the truncated C-terminal portion of Bid. FEBS J..

[234] Ghibelli L., Diederich M. (2010). multistep and multitask Bax activation. Mitochondria Res. Soc..

[235] Hsu Y.T., Wolter K.G., Youle R.J. (1997). Cytosol-to-membrane redistribution of Bax and Bcl-X(L) during apoptosis. Proc. Natl. Acad. Sci. USA.

[236] Wolter K.G., Hsu Y.T., Smith C.L., Nechushtan A., Xi X.G., Youle R.J. (1997). Movement of Bax from the cytosol to mitochondria during apoptosis. J. Cell Biol..

[237] Kuwana T., Newmeyer D.D. (2003). Bcl-2-family proteins and the role of mitochondria in apoptosis. Curr. Opin. Cell Biol..

[238] Li B., Dou Q.P. (2000). Bax degradation by the ubiquitin/proteasome-dependent pathway: Involvement in tumor survival and progression. Proc. Natl. Acad. Sci. USA.

[239] Liu F.T., Agrawal S.G., Gribben J.G., Ye H., Du M.Q., Newland A.C., Jia L. (2008). Bortezomib blocks Bax degradation in malignant B cells during treatment with TRAIL. Blood.

[240] Chang Y.C., Lee Y.S., Tejima T., Tanaka K., Omura S., Heintz N.H., Mitsui Y., Magae J. (1998). mdm2 and bax, downstream mediators of the p53 response, are degraded by the ubiquitin-proteasome pathway. Cell Growth Differ..

[241] Benard G., Neutzner A., Peng G., Wang C., Livak F., Youle R.J., Karbowski M. (2010). IBRDC2, an IBR-type E3 ubiquitin ligase, is a regulatory factor for Bax and apoptosis activation. EMBO J..

[242] Johnson B.N., Berger A.K., Cortese G.P., Lavoie M.J. (2012). The ubiquitin E3 ligase parkin regulates the proapoptotic function of Bax. Proc. Natl. Acad. Sci. USA.

[243] Srinivasula S.M., Ashwell J.D. (2008). IAPs: What’s in a name?. Mol. Cell.

[244] Schimmer A.D. (2004). Inhibitor of apoptosis proteins: Translating basic knowledge into clinical practice. Cancer Res..

[245] Rothe M., Pan M.G., Henzel W.J., Ayres T.M., Goeddel D.V. (1995). The TNFR2-TRAF signaling complex contains two novel proteins related to baculoviral inhibitor of apoptosis proteins. Cell.

[246] Shu H.B., Takeuchi M., Goeddel D.V. (1996). The tumor necrosis factor receptor 2 signal transducers TRAF2 and c-IAP1 are components of the tumor necrosis factor receptor 1 signaling complex. Proc. Natl. Acad. Sci. USA.

[247] Varfolomeev E., Blankenship J.W., Wayson S.M., Fedorova A.V., Kayagaki N., Garg P., Zobel K., Dynek J.N., Elliott L.O., Wallweber H.J. (2007). IAP antagonists induce autoubiquitination of c-IAPs, NF-kappaB activation, and TNFalpha-dependent apoptosis. Cell.

[248] Gyrd-Hansen M., Meier P. (2010). IAPs: From caspase inhibitors to modulators of NF-kappaB, inflammation and cancer. Nat. Rev. Cancer.

[249] Vallabhapurapu S., Matsuzawa A., Zhang W., Tseng P.H., Keats J.J., Wang H., Vignali D.A., Bergsagel P.L., Karin M. (2008). Nonredundant and complementary functions of TRAF2 and TRAF3 in a ubiquitination cascade that activates NIK-dependent alternative NF-kappaB signaling. Nat. Immunol..

[250] Bertrand M.J., Milutinovic S., Dickson K.M., Ho W.C., Boudreault A., Durkin J., Gillard J.W., Jaquith J.B., Morris S.J., Barker P.A. (2008). cIAP1 and cIAP2 facilitate cancer cell survival by functioning as E3 ligases that promote RIP1 ubiquitination. Mol. Cell.

[251] Varfolomeev E., Goncharov T., Fedorova A.V., Dynek J.N., Zobel K., Deshayes K., Fairbrother W.J., Vucic D. (2008). c-IAP1 and c-IAP2 are critical mediators of tumor necrosis factor alpha (TNFalpha)-induced NF-kappaB activation. J. Biol. Chem..

[252] Vince J.E., Wong W.W., Khan N., Feltham R., Chau D., Ahmed A.U., Benetatos C.A., Chunduru S.K., Condon S.M., McKinlay M. (2007). IAP antagonists target cIAP1 to induce TNFalpha-dependent apoptosis. Cell.

[253] Gaither A., Porter D., Yao Y., Borawski J., Yang G., Donovan J., Sage D., Slisz J., Tran M., Straub C. (2007). A Smac mimetic rescue screen reveals roles for inhibitor of apoptosis proteins in tumor necrosis factor-alpha signaling. Cancer Res..

[254] Bonizzi G., Karin M. (2004). The two NF-kappaB activation pathways and their role in innate and adaptive immunity. Trends Immunol..

[255] Dubrez L., Berthelet J., Glorian V. (2013). IAP proteins as targets for drug development in oncology. Onco Targets Ther..

[256] Danson S., Dean E., Dive C., Ranson M. (2007). IAPs as a target for anticancer therapy. Curr. Cancer Drug Targets.

[257] Hegde R., Srinivasula S.M., Zhang Z., Wassell R., Mukattash R., Cilenti L., DuBois G., Lazebnik Y., Zervos A.S., Fernandes-Alnemri T. (2002). Identification of Omi/HtrA2 as a mitochondrial apoptotic serine protease that disrupts inhibitor of apoptosis protein-caspase interaction. J. Biol. Chem..

[258] Elhasid R., Sahar D., Merling A., Zivony Y., Rotem A., Ben-Arush M., Izraeli S., Bercovich D., Larisch S. (2004). Mitochondrial pro-apoptotic ARTS protein is lost in the majority of acute lymphoblastic leukemia patients. Oncogene.

[259] Wing J.P., Schwartz L.M., Nambu J.R. (2001). The RHG motifs of Drosophila Reaper and Grim are important for their distinct cell death-inducing abilities. Mech. Dev..

[260] Jost P.J., Vucic D. (2019). Regulation of Cell Death and Immunity by XIAP. Cold Spring Harb. Perspect. Biol..

[261] Steller H. (2008). Regulation of apoptosis in Drosophila. Cell Death Differ..

[262] Kornbluth S., White K. (2005). Apoptosis in Drosophila: Neither fish nor fowl (nor man, nor worm). J. Cell Sci..

[263] Du C., Fang M., Li Y., Li L., Wang X. (2000). Smac, a mitochondrial protein that promotes cytochrome c-dependent caspase activation by eliminating IAP inhibition. Cell.

[264] Verhagen A.M., Ekert P.G., Pakusch M., Silke J., Connolly L.M., Reid G.E., Moritz R.L., Simpson R.J., Vaux D.L. (2000). Identification of DIABLO, a mammalian protein that promotes apoptosis by binding to and antagonizing IAP proteins. Cell.

[265] Yin W., Cheepala S., Clifford J.L. (2006). Identification of a novel splice variant of X-linked inhibitor of apoptosis-associated factor 1. Biochem. Biophys. Res. Commun..

[266] Larisch S., Yi Y., Lotan R., Kerner H., Eimerl S., Tony Parks W., Gottfried Y., Birkey Reffey S., de Caestecker M.P., Danielpour D. (2000). A novel mitochondrial septin-like protein, ARTS, mediates apoptosis dependent on its P-loop motif. Nat. Cell Biol..

[267] van Loo G., van Gurp M., Depuydt B., Srinivasula S.M., Rodriguez I., Alnemri E.S., Gevaert K., Vandekerckhove J., Declercq W., Vandenabeele P. (2002). The serine protease Omi/HtrA2 is released from mitochondria during apoptosis. Omi interacts with caspase-inhibitor XIAP and induces enhanced caspase activity. Cell Death Differ..

[268] Leaman D.W., Chawla-Sarkar M., Vyas K., Reheman M., Tamai K., Toji S., Borden E.C. (2002). Identification of X-linked inhibitor of apoptosis-associated factor-1 as an interferon-stimulated gene that augments TRAIL Apo2L-induced apoptosis. J. Biol. Chem..

[269] Srinivasula S.M., Hegde R., Saleh A., Datta P., Shiozaki E., Chai J., Lee R.A., Robbins P.D., Fernandes-Alnemri T., Shi Y. (2001). A conserved XIAP-interaction motif in caspase-9 and Smac/DIABLO regulates caspase activity and apoptosis. Nature.

[270] Shi Y. (2002). A conserved tetrapeptide motif: Potentiating apoptosis through IAP-binding. Cell Death Differ..

[271] Goyal L., McCall K., Agapite J., Hartwieg E., Steller H. (2000). Induction of apoptosis by Drosophila reaper, hid and grim through inhibition of IAP function. EMBO J..

[272] Wu G., Chai J., Suber T.L., Wu J.W., Du C., Wang X., Shi Y. (2000). Structural basis of IAP recognition by Smac/DIABLO. Nature.

[273] Grether M.E., Abrams J.M., Agapite J., White K., Steller H. (1995). The head involution defective gene of Drosophila melanogaster functions in programmed cell death. Genes Dev..

[274] White K., Grether M.E., Abrams J.M., Young L., Farrell K., Steller H. (1994). Genetic control of programmed cell death in Drosophila. Science.

[275] Hay B.A., Wassarman D.A., Rubin G.M. (1995). Drosophila homologs of baculovirus inhibitor of apoptosis proteins function to block cell death. Cell.

[276] Wang S.L., Hawkins C.J., Yoo S.J., Muller H.A., Hay B.A. (1999). The Drosophila caspase inhibitor DIAP1 is essential for cell survival and is negatively regulated by HID. Cell.

[277] Burri L., Strahm Y., Hawkins C.J., Gentle I.E., Puryer M.A., Verhagen A., Callus B., Vaux D., Lithgow T. (2005). Mature DIABLO/Smac is produced by the IMP protease complex on the mitochondrial inner membrane. Mol. Biol. Cell.

[278] Rodriguez J., Lazebnik Y. (1999). Caspase-9 and APAF-1 form an active holoenzyme. Genes Dev..

[279] Adrain C., Creagh E.M., Martin S.J. (2001). Apoptosis-associated release of Smac/DIABLO from mitochondria requires active caspases and is blocked by Bcl-2. EMBO J..

[280] Li L., Thomas R.M., Suzuki H., De Brabander J.K., Wang X., Harran P.G. (2004). A small molecule Smac mimic potentiates TRAIL- and TNFalpha-mediated cell death. Science.

[281] Chai J., Du C., Wu J.W., Kyin S., Wang X., Shi Y. (2000). Structural and biochemical basis of apoptotic activation by Smac/DIABLO. Nature.

[282] Yang Q.H., Du C. (2004). Smac/DIABLO selectively reduces the levels of c-IAP1 and c-IAP2 but not that of XIAP and livin in HeLa cells. J. Biol. Chem..

[283] Creagh E.M., Murphy B.M., Duriez P.J., Duckett C.S., Martin S.J. (2004). Smac/Diablo antagonizes ubiquitin ligase activity of inhibitor of apoptosis proteins. J. Biol. Chem..

[284] Mandel-Gutfreund Y., Kosti I., Larisch S. (2011). ARTS, the unusual septin: Structural and functional aspects. Biol. Chem..

[285] Lakhani S.A., Masud A., Kuida K., Porter G.A., Booth C.J., Mehal W.Z., Inayat I., Flavell R.A. (2006). Caspases 3 and 7: Key mediators of mitochondrial events of apoptosis. Science.

[286] Braun T., Dar S., Vorobiov D., Lindenboim L., Dascal N., Stein R. (2003). Expression of Bcl-x(S) in Xenopus oocytes induces BH3-dependent and caspase-dependent cytochrome c release and apoptosis. Mol. Cancer Res..

[287] Gao C.F., Ren S., Zhang L., Nakajima T., Ichinose S., Hara T., Koike K., Tsuchida N. (2001). Caspase-dependent cytosolic release of cytochrome c and membrane translocation of Bax in p53-induced apoptosis. Exp. Cell Res..

[288] Ho A.T., Zacksenhaus E. (2004). Splitting the apoptosome. Cell Cycle.

[289] Shawgo M.E., Shelton S.N., Robertson J.D. (2008). Caspase-mediated Bak activation and cytochrome c release during intrinsic apoptotic cell death in Jurkat cells. J. Biol. Chem..

[290] Manns J., Daubrawa M., Driessen S., Paasch F., Hoffmann N., Loffler A., Lauber K., Dieterle A., Alers S., Iftner T. (2011). Triggering of a novel intrinsic apoptosis pathway by the kinase inhibitor staurosporine: Activation of caspase-9 in the absence of Apaf-1. FASEB J..

[291] Morizane Y., Honda R., Fukami K., Yasuda H. (2005). X-linked inhibitor of apoptosis functions as ubiquitin ligase toward mature caspase-9 and cytosolic Smac/DIABLO. J. Biochem..

[292] Wilkinson J.C., Wilkinson A.S., Galban S., Csomos R.A., Duckett C.S. (2008). Apoptosis-inducing factor is a target for ubiquitination through interaction with XIAP. Mol. Cell Biol..

[293] Dumetier B., Zadoroznyj A., Dubrez L. (2020). IAP-Mediated Protein Ubiquitination in Regulating Cell Signaling. Cells.

[294] MacFarlane M., Merrison W., Bratton S.B., Cohen G.M. (2002). Proteasome-mediated degradation of Smac during apoptosis: XIAP promotes Smac ubiquitination in vitro. J. Biol. Chem..

[295] Qin S., Yang C., Zhang B., Li X., Sun X., Li G., Zhang J., Xiao G., Gao X., Huang G. (2016). XIAP inhibits mature Smac-induced apoptosis by degrading it through ubiquitination in NSCLC. Int. J. Oncol..

[296] Kemeny S., Dery D., Loboda Y., Rovner M., Lev T., Zuri D., Finberg J.P.M., Larisch S. (2012). Parkin promotes degradation of the mitochondrial pro-apoptotic ARTS protein. PLoS ONE.

[297] Edison N., Reingewertz T.H., Gottfried Y., Lev T., Zuri D., Maniv I., Carp M.J., Shalev G., Friedler A., Larisch S. (2012). Peptides Mimicking the Unique ARTS-XIAP Binding Site Promote Apoptotic Cell Death in Cultured Cancer Cells. Clin. Cancer Res..

[298] Garrison J.B., Correa R.G., Gerlic M., Yip K.W., Krieg A., Tamble C.M., Shi R., Welsh K., Duggineni S., Huang Z. (2011). ARTS and Siah collaborate in a pathway for XIAP degradation. Mol. Cell.

[299] Garcia-Fernandez M., Kissel H., Brown S., Gorenc T., Schile A.J., Rafii S., Larisch S., Steller H. (2010). Sept4/ARTS is required for stem cell apoptosis and tumor suppression. Genes Dev..

[300] Samuel T., Welsh K., Lober T., Togo S.H., Zapata J.M., Reed J.C. (2006). Distinct BIR domains of cIAP1 mediate binding to and ubiquitination of tumor necrosis factor receptor-associated factor 2 and second mitochondrial activator of caspases. J. Biol. Chem..

[301] Tenev T., Bianchi K., Darding M., Broemer M., Langlais C., Wallberg F., Zachariou A., Lopez J., MacFarlane M., Cain K. (2011). The Ripoptosome, a signaling platform that assembles in response to genotoxic stress and loss of IAPs. Mol. Cell.

[302] Li X., Yang Y., Ashwell J.D. (2002). TNF-RII and c-IAP1 mediate ubiquitination and degradation of TRAF2. Nature.

[303] Matsuzawa A., Tseng P.H., Vallabhapurapu S., Luo J.L., Zhang W., Wang H., Vignali D.A., Gallagher E., Karin M. (2008). Essential cytoplasmic translocation of a cytokine receptor-assembled signaling complex. Science.

[304] Tseng P.H., Matsuzawa A., Zhang W., Mino T., Vignali D.A., Karin M. (2010). Different modes of ubiquitination of the adaptor TRAF3 selectively activate the expression of type I interferons and proinflammatory cytokines. Nat. Immunol..

[305] Ma L., Huang Y., Song Z., Feng S., Tian X., Du W., Qiu X., Heese K., Wu M. (2006). Livin promotes Smac/DIABLO degradation by ubiquitin-proteasome pathway. Cell Death Differ..

[306] Kim J.B., Kim S.Y., Kim B.M., Lee H., Kim I., Yun J., Jo Y., Oh T., Jo Y., Chae H.D. (2013). Identification of a novel anti-apoptotic E3 ubiquitin ligase that ubiquitinates antagonists of inhibitor of apoptosis proteins SMAC, HtrA2, and ARTS. J. Biol. Chem..

[307] Ciechanover A. (1994). The ubiquitin-proteasome proteolytic pathway. Cell.

[308] Jana N.R. (2012). Protein homeostasis and aging: Role of ubiquitin protein ligases. Neurochem. Int..

[309] Jarpe M.B., Widmann C., Knall C., Schlesinger T.K., Gibson S., Yujiri T., Fanger G.R., Gelfand E.W., Johnson G.L. (1998). Anti-apoptotic versus pro-apoptotic signal transduction: Checkpoints and stop signs along the road to death. Oncogene.

[310] Opferman J.T., Kothari A. (2018). Anti-apoptotic BCL-2 family members in development. Cell Death Differ..

[311] Yang E., Korsmeyer S.J. (1996). Molecular thanatopsis: A discourse on the BCL2 family and cell death. Blood.

[312] Prehn J.H., Bindokas V.P., Marcuccilli C.J., Krajewski S., Reed J.C., Miller R.J. (1994). Regulation of neuronal Bcl2 protein expression and calcium homeostasis by transforming growth factor type beta confers wide-ranging protection on rat hippocampal neurons. Proc. Natl. Acad. Sci. USA.

[313] Fricker L.D. (2020). Proteasome Inhibitor Drugs. Annu. Rev. Pharmacol. Toxicol..

[314] Gandolfi S., Laubach J.P., Hideshima T., Chauhan D., Anderson K.C., Richardson P.G. (2017). The proteasome and proteasome inhibitors in multiple myeloma. Cancer Metastasis Rev..

[315] Zhang J., Yang P.L., Gray N.S. (2009). Targeting cancer with small molecule kinase inhibitors. Nat. Rev. Cancer.

[316] Huang X., Dixit V.M. (2016). Drugging the undruggables: Exploring the ubiquitin system for drug development. Cell Res..

[317] Narayanan S., Cai C.Y., Assaraf Y.G., Guo H.Q., Cui Q., Wei L., Huang J.J., Ashby C.R., Chen Z.S. (2020). Targeting the ubiquitin-proteasome pathway to overcome anti-cancer drug resistance. Drug Resist. Updates.

[318] Crew A.P., Raina K., Dong H., Qian Y., Wang J., Vigil D., Serebrenik Y.V., Hamman B.D., Morgan A., Ferraro C. (2018). Identification and Characterization of Von Hippel-Lindau-Recruiting Proteolysis Targeting Chimeras (PROTACs) of TANK-Binding Kinase 1. J. Med. Chem..

[319] Kane R.C., Bross P.F., Farrell A.T., Pazdur R. (2003). Velcade: U.S. FDA approval for the treatment of multiple myeloma progressing on prior therapy. Oncologist.

[320] Richardson P.G., Mitsiades C., Hideshima T., Anderson K.C. (2006). Bortezomib: Proteasome inhibition as an effective anticancer therapy. Annu. Rev. Med..

[321] Goldberg A.L. (2012). Development of proteasome inhibitors as research tools and cancer drugs. J. Cell Biol..

[322] Goldberg A.L., Ghobrial I.M., Richardson P.G., Anderson K.C. (2011). Bortezomib’s Scientific Origins and Its Tortuous Path to the Clinic. Bortezomib in the Treatment of Multiple Myeloma.

[323] Reinstein E., Ciechanover A. (2006). Narrative review: Protein degradation and human diseases: The ubiquitin connection. Ann. Intern. Med..

[324] Qin J.Z., Ziffra J., Stennett L., Bodner B., Bonish B.K., Chaturvedi V., Bennett F., Pollock P.M., Trent J.M., Hendrix M.J. (2005). Proteasome inhibitors trigger NOXA-mediated apoptosis in melanoma and myeloma cells. Cancer Res..

[325] Kortuem K.M., Stewart A.K. (2013). Carfilzomib. Blood.

[326] Kuhn D.J., Chen Q., Voorhees P.M., Strader J.S., Shenk K.D., Sun C.M., Demo S.D., Bennett M.K., van Leeuwen F.W., Chanan-Khan A.A. (2007). Potent activity of carfilzomib, a novel, irreversible inhibitor of the ubiquitin-proteasome pathway, against preclinical models of multiple myeloma. Blood.

[327] Parlati F., Lee S.J., Aujay M., Suzuki E., Levitsky K., Lorens J.B., Micklem D.R., Ruurs P., Sylvain C., Lu Y. (2009). Carfilzomib can induce tumor cell death through selective inhibition of the chymotrypsin-like activity of the proteasome. Blood.

[328] Al-Salama Z.T., Garnock-Jones K.P., Scott L.J. (2017). Ixazomib: A Review in Relapsed and/or Refractory Multiple Myeloma. Target. Oncol..

[329] Manasanch E.E., Orlowski R.Z. (2017). Proteasome inhibitors in cancer therapy. Nat. Rev. Clin. Oncol..

[330] Chauhan D., Tian Z., Zhou B., Kuhn D., Orlowski R., Raje N., Richardson P., Anderson K.C. (2011). In vitro and in vivo selective antitumor activity of a novel orally bioavailable proteasome inhibitor MLN9708 against multiple myeloma cells. Clin. Cancer Res.

[331] Piva R., Ruggeri B., Williams M., Costa G., Tamagno I., Ferrero D., Giai V., Coscia M., Peola S., Massaia M. (2008). CEP-18770: A novel, orally active proteasome inhibitor with a tumor-selective pharmacologic profile competitive with bortezomib. Blood.

[332] Vogl D.T., Martin T.G., Vij R., Hari P., Mikhael J.R., Siegel D., Wu K.L., Delforge M., Gasparetto C. (2017). Phase I/II study of the novel proteasome inhibitor delanzomib (CEP-18770) for relapsed and refractory multiple myeloma. Leuk Lymphoma.

[333] Chauhan D., Catley L., Li G., Podar K., Hideshima T., Velankar M., Mitsiades C., Mitsiades N., Yasui H., Letai A. (2005). A novel orally active proteasome inhibitor induces apoptosis in multiple myeloma cells with mechanisms distinct from Bortezomib. Cancer Cell.

[334] Shah J., Usmani S., Stadtmauer E.A., Rifkin R.M., Berenson J.R., Berdeja J.G., Lyons R.M., Klippel Z., Chang Y.L., Niesvizky R. (2019). Oprozomib, pomalidomide, and Dexamethasone in Patients With Relapsed and/or Refractory Multiple Myeloma. Clin. Lymphoma Myeloma Leuk.

[335] Zhu H., Wang T., Xin Z., Zhan Y., Gu G., Li X., Wang X., Yang S., Liu C. (2019). An oral second-generation proteasome inhibitor oprozomib significantly inhibits lung cancer in a p53 independent manner in vitro. Acta Biochim. Biophys. Sin..

[336] Potts B.C., Albitar M.X., Anderson K.C., Baritaki S., Berkers C., Bonavida B., Chandra J., Chauhan D., Cusack J.C., Fenical W. (2011). Marizomib, a proteasome inhibitor for all seasons: Preclinical profile and a framework for clinical trials. Curr. Cancer Drug Targets.

[337] Zheng Z., Liu T., Zheng J., Hu J. (2017). Clarifying the molecular mechanism associated with carfilzomib resistance in human multiple myeloma using microarray gene expression profile and genetic interaction network. Onco Targets Ther..

[338] Chene P. (2003). Inhibiting the p53-MDM2 interaction: An important target for cancer therapy. Nat. Rev. Cancer.

[339] Quesnel B., Preudhomme C., Oscier D., Lepelley P., Collyn-d’Hooghe M., Facon T., Zandecki M., Fenaux P. (1994). Over-expression of the MDM2 gene is found in some cases of haematological malignancies. Br. J. Haematol..

[340] Vassilev L.T., Vu B.T., Graves B., Carvajal D., Podlaski F., Filipovic Z., Kong N., Kammlott U., Lukacs C., Klein C. (2004). In vivo activation of the p53 pathway by small-molecule antagonists of MDM2. Science.

[341] Ding Q., Zhang Z., Liu J.J., Jiang N., Zhang J., Ross T.M., Chu X.J., Bartkovitz D., Podlaski F., Janson C. (2013). Discovery of RG7388, a potent and selective p53-MDM2 inhibitor in clinical development. J. Med. Chem..

[342] Zhang Z., Chu X.J., Liu J.J., Ding Q., Zhang J., Bartkovitz D., Jiang N., Karnachi P., So S.S., Tovar C. (2014). Discovery of Potent and Orally Active p53-MDM2 Inhibitors RO5353 and RO2468 for Potential Clinical Development. ACS Med. Chem. Lett..

[343] Vu B., Wovkulich P., Pizzolato G., Lovey A., Ding Q., Jiang N., Liu J.J., Zhao C., Glenn K., Wen Y. (2013). Discovery of RG7112: A Small-Molecule MDM2 Inhibitor in Clinical Development. ACS Med. Chem. Lett..

[344] Ray-Coquard I., Blay J.Y., Italiano A., Le Cesne A., Penel N., Zhi J., Heil F., Rueger R., Graves B., Ding M. (2012). Effect of the MDM2 antagonist RG7112 on the P53 pathway in patients with MDM2-amplified, well-differentiated or dedifferentiated liposarcoma: An exploratory proof-of-mechanism study. Lancet Oncol..

[345] Fang Y., Liao G., Yu B. (2020). Small-molecule MDM2/X inhibitors and PROTAC degraders for cancer therapy: Advances and perspectives. Acta Pharm. Sin. B.

[346] Nguyen D., Liao W., Zeng S.X., Lu H. (2017). Reviving the guardian of the genome: Small molecule activators of p53. Pharmacol. Ther..

[347] Ladds M., Lain S. (2019). Small molecule activators of the p53 response. J. Mol. Cell Biol..

[348] Konopleva M., Martinelli G., Daver N., Papayannidis C., Wei A., Higgins B., Ott M., Mascarenhas J., Andreeff M. (2020). MDM2 inhibition: An important step forward in cancer therapy. Leukemia.

[349] Fischer K., Al-Sawaf O., Fink A.M., Dixon M., Bahlo J., Warburton S., Kipps T.J., Weinkove R., Robinson S., Seiler T. (2017). Venetoclax and obinutuzumab in chronic lymphocytic leukemia. Blood.

[350] Mihalyova J., Jelinek T., Growkova K., Hrdinka M., Simicek M., Hajek R. (2018). Venetoclax: A new wave in hematooncology. Exp. Hematol.

[351] Pollyea D.A., Stevens B.M., Jones C.L., Winters A., Pei S., Minhajuddin M., D’Alessandro A., Culp-Hill R., Riemondy K.A., Gillen A.E. (2018). Venetoclax with azacitidine disrupts energy metabolism and targets leukemia stem cells in patients with acute myeloid leukemia. Nat. Med..

[352] Rogers K.A., Huang Y., Ruppert A.S., Awan F.T., Heerema N.A., Hoffman C., Lozanski G., Maddocks K.J., Moran M.E., Reid M.A. (2018). Phase 1b study of obinutuzumab, ibrutinib, and venetoclax in relapsed and refractory chronic lymphocytic leukemia. Blood.

[353] Stilgenbauer S., Eichhorst B., Schetelig J., Coutre S., Seymour J.F., Munir T., Puvvada S.D., Wendtner C.-M., Roberts A.W., Jurczak W. (2016). Venetoclax in relapsed or refractory chronic lymphocytic leukaemia with 17p deletion: A multicentre, open-label, phase 2 study. Lancet Oncol..

[354] Shahar N., Larisch S. (2020). Inhibiting the inhibitors: Targeting anti-apoptotic proteins in cancer and therapy resistance. Drug Resist. Updates.

[355] Crews C.M. (2010). Targeting the undruggable proteome: The small molecules of my dreams. Chem. Biol..

[356] Lai A.C., Crews C.M. (2017). Induced protein degradation: An emerging drug discovery paradigm. Nat. Rev. Drug Discov..

[357] Overington J.P., Al-Lazikani B., Hopkins A.L. (2006). How many drug targets are there?. Nat. Rev. Drug Discov..

[358] Sakamoto K.M., Kim K.B., Kumagai A., Mercurio F., Crews C.M., Deshaies R.J. (2001). Protacs: Chimeric molecules that target proteins to the Skp1-Cullin-F box complex for ubiquitination and degradation. Proc. Natl. Acad. Sci. USA.

[359] Sun X., Gao H., Yang Y., He M., Wu Y., Song Y., Tong Y., Rao Y. (2019). PROTACs: Great opportunities for academia and industry. Signal. Transduct. Target. Ther..

[360] Sun B., Fiskus W., Qian Y., Rajapakshe K., Raina K., Coleman K.G., Crew A.P., Shen A., Saenz D.T., Mill C.P. (2018). BET protein proteolysis targeting chimera (PROTAC) exerts potent lethal activity against mantle cell lymphoma cells. Leukemia.

[361] He Y., Koch R., Budamagunta V., Zhang P., Zhang X., Khan S., Thummuri D., Ortiz Y.T., Zhang X., Lv D. (2020). DT2216-a Bcl-xL-specific degrader is highly active against Bcl-xL-dependent T cell lymphomas. J. Hematol. Oncol..

[362] Khan S., Zhang X., Lv D., Zhang Q., He Y., Zhang P., Liu X., Thummuri D., Yuan Y., Wiegand J.S. (2019). A selective BCL-XL PROTAC degrader achieves safe and potent antitumor activity. Nat. Med..

[363] Zhang X., Liu X., Zhou D., Zheng G. (2020). Targeting anti-apoptotic BCL-2 family proteins for cancer treatment. Future Med. Chem..

[364] Wang Z., He N., Guo Z., Niu C., Song T., Guo Y., Cao K., Wang A., Zhu J., Zhang X. (2019). Proteolysis Targeting Chimeras for the Selective Degradation of Mcl-1/Bcl-2 Derived from Nonselective Target Binding Ligands. J. Med. Chem..

[365] Papatzimas J.W., Gorobets E., Maity R., Muniyat M.I., MacCallum J.L., Neri P., Bahlis N.J., Derksen D.J. (2019). From Inhibition to Degradation: Targeting the Antiapoptotic Protein Myeloid Cell Leukemia 1 (MCL1). J. Med. Chem..

[366] Yang Y., Fang S., Jensen J.P., Weissman A.M., Ashwell J.D. (2000). Ubiquitin protein ligase activity of IAPs and their degradation in proteasomes in response to apoptotic stimuli. Science.

[367] Esposito I., Kleeff J., Abiatari I., Shi X., Giese N., Bergmann F., Roth W., Friess H., Schirmacher P. (2007). Overexpression of cellular inhibitor of apoptosis protein 2 is an early event in the progression of pancreatic cancer. J. Clin. Pathol..

[368] Che X., Yang D., Zong H., Wang J., Li X., Chen F., Chen X., Song X. (2012). Nuclear cIAP1 overexpression is a tumor stage- and grade-independent predictor of poor prognosis in human bladder cancer patients. Urol Oncol..

[369] Oost T.K., Sun C., Armstrong R.C., Al-Assaad A.S., Betz S.F., Deckwerth T.L., Ding H., Elmore S.W., Meadows R.P., Olejniczak E.T. (2004). Discovery of potent antagonists of the antiapoptotic protein XIAP for the treatment of cancer. J. Med. Chem..

[370] Bergmann A., Yang A.Y., Srivastava M. (2003). Regulators of IAP function: Coming to grips with the grim reaper. Curr. Opin. Cell Biol..

[371] Sun H., Nikolovska-Coleska Z., Yang C.Y., Qian D., Lu J., Qiu S., Bai L., Peng Y., Cai Q., Wang S. (2008). Design of small-molecule peptidic and nonpeptidic Smac mimetics. Acc. Chem. Res..

[372] Sun H., Nikolovska-Coleska Z., Yang C.Y., Xu L., Liu M., Tomita Y., Pan H., Yoshioka Y., Krajewski K., Roller P.P. (2004). Structure-based design of potent, conformationally constrained Smac mimetics. J. Am. Chem. Soc..

[373] Corti A., Milani M., Lecis D., Seneci P., de Rosa M., Mastrangelo E., Cossu F. (2018). Structure-based design and molecular profiling of Smac-mimetics selective for cellular IAPs. FEBS J..

[374] Welsh K., Milutinovic S., Ardecky R.J., Gonzalez-Lopez M., Ganji S.R., Teriete P., Finlay D., Riedl S., Matsuzawa S., Pinilla C. (2016). Characterization of Potent SMAC Mimetics that Sensitize Cancer Cells to TNF Family-Induced Apoptosis. PLoS ONE.

[375] Liu Z., Sun C., Olejniczak E.T., Meadows R.P., Betz S.F., Oost T., Herrmann J., Wu J.C., Fesik S.W. (2000). Structural basis for binding of Smac/DIABLO to the XIAP BIR3 domain. Nature.

[376] Sharma S.K., Straub C., Zawel L. (2006). Development of Peptidomimetics Targeting IAPs. Int. J. Pept. Res. Ther..

[377] Zobel K., Wang L., Varfolomeev E., Franklin M.C., Elliott L.O., Wallweber H.J., Okawa D.C., Flygare J.A., Vucic D., Fairbrother W.J. (2006). Design, synthesis, and biological activity of a potent Smac mimetic that sensitizes cancer cells to apoptosis by antagonizing IAPs. ACS Chem. Biol..

[378] Darding M., Feltham R., Tenev T., Bianchi K., Benetatos C., Silke J., Meier P. (2011). Molecular determinants of Smac mimetic induced degradation of cIAP1 and cIAP2. Cell Death Differ..

[379] He S., Wang L., Miao L., Wang T., Du F., Zhao L., Wang X. (2009). Receptor interacting protein kinase-3 determines cellular necrotic response to TNF-alpha. Cell.

[380] Wang L., Du F., Wang X. (2008). TNF-alpha induces two distinct caspase-8 activation pathways. Cell.

[381] Barnhart B.C., Peter M.E. (2003). The TNF receptor 1: A split personality complex. Cell.

[382] Schutze S., Tchikov V., Schneider-Brachert W. (2008). Regulation of TNFR1 and CD95 signalling by receptor compartmentalization. Nat. Rev. Mol. Cell Biol..

[383] Petersen S.L., Wang L., Yalcin-Chin A., Li L., Peyton M., Minna J., Harran P., Wang X. (2007). Autocrine TNFalpha signaling renders human cancer cells susceptible to Smac-mimetic-induced apoptosis. Cancer Cell.

[384] Darding M., Meier P. (2012). IAPs: Guardians of RIPK1. Cell Death Differ..

[385] Derakhshan A., Chen Z., Van Waes C. (2017). Therapeutic Small Molecules Target Inhibitor of Apoptosis Proteins in Cancers with Deregulation of Extrinsic and Intrinsic Cell Death Pathways. Clin. Cancer Res..

[386] Collart M.A., Baeuerle P., Vassalli P. (1990). Regulation of tumor necrosis factor alpha transcription in macrophages: Involvement of four kappa B-like motifs and of constitutive and inducible forms of NF-kappa B. Mol. Cell Biol..

[387] Kondylis V., Kumari S., Vlantis K., Pasparakis M. (2017). The interplay of IKK, NF-kappaB and RIPK1 signaling in the regulation of cell death, tissue homeostasis and inflammation. Immunol. Rev..

[388] Condon S.M., Mitsuuchi Y., Deng Y., LaPorte M.G., Rippin S.R., Haimowitz T., Alexander M.D., Kumar P.T., Hendi M.S., Lee Y.H. (2014). Birinapant, a smac-mimetic with improved tolerability for the treatment of solid tumors and hematological malignancies. J. Med. Chem..

[389] Amaravadi R.K., Schilder R.J., Martin L.P., Levin M., Graham M.A., Weng D.E., Adjei A.A. (2015). A Phase I Study of the SMAC-Mimetic Birinapant in Adults with Refractory Solid Tumors or Lymphoma. Mol. Cancer Ther..

[390] Benetatos C.A., Mitsuuchi Y., Burns J.M., Neiman E.M., Condon S.M., Yu G., Seipel M.E., Kapoor G.S., Laporte M.G., Rippin S.R. (2014). Birinapant (TL32711), a bivalent SMAC mimetic, targets TRAF2-associated cIAPs, abrogates TNF-induced NF-kappaB activation, and is active in patient-derived xenograft models. Mol. Cancer Ther..

[391] Finlay D., Teriete P., Vamos M., Cosford N.D.P., Vuori K. (2017). Inducing death in tumor cells: Roles of the inhibitor of apoptosis proteins. F1000Research.

[392] Maas C., Tromp J.M., van Laar J., Thijssen R., Elias J.A., Malara A., Krippner-Heidenreich A., Silke J., van Oers M.H., Eldering E. (2013). CLL cells are resistant to smac mimetics because of an inability to form a ripoptosome complex. Cell Death Dis..

[393] Petersen S.L., Peyton M., Minna J.D., Wang X. (2010). Overcoming cancer cell resistance to Smac mimetic induced apoptosis by modulating cIAP-2 expression. Proc. Natl. Acad. Sci. USA.

[394] Shekhar T.M., Burvenich I.J.G., Harris M.A., Rigopoulos A., Zanker D., Spurling A., Parker B.S., Walkley C.R., Scott A.M., Hawkins C.J. (2019). Smac mimetics LCL161 and GDC-0152 inhibit osteosarcoma growth and metastasis in mice. BMC Cancer.

[395] Chen Z., Chen J., Liu H., Dong W., Huang X., Yang D., Hou J., Zhang X. (2018). The SMAC Mimetic APG-1387 Sensitizes Immune-Mediated Cell Apoptosis in Hepatocellular Carcinoma. Front. Pharmacol..

[396] Scheurer M.J.J., Seher A., Steinacker V., Linz C., Hartmann S., Kubler A.C., Muller-Richter U.D.A., Brands R.C. (2019). Targeting inhibitors of apoptosis in oral squamous cell carcinoma in vitro. J. Craniomaxillofac. Surg..

[397] Mamriev D., Abbas R., Klingler F.M., Kagan J., Kfir N., Donald A., Weidenfeld K., Sheppard D.W., Barkan D., Larisch S. (2020). A small-molecule ARTS mimetic promotes apoptosis through degradation of both XIAP and Bcl-2. Cell Death Dis..

[398] Naito M., Ohoka N., Shibata N. (2019). SNIPERs-Hijacking IAP activity to induce protein degradation. Drug Discov. Today Technol.

[399] Ohoka N., Ujikawa O., Shimokawa K., Sameshima T., Shibata N., Hattori T., Nara H., Cho N., Naito M. (2019). Different Degradation Mechanisms of Inhibitor of Apoptosis Proteins (IAPs) by the Specific and Nongenetic IAP-Dependent Protein Eraser (SNIPER). Chem. Pharm. Bull..

[400] Ishikawa M., Tomoshige S., Demizu Y., Naito M. (2020). Selective Degradation of Target Proteins by Chimeric Small-Molecular Drugs, PROTACs and SNIPERs. Pharmaceuticals.

[401] Ma Z., Ji Y., Yu Y., Liang D. (2021). Specific non-genetic IAP-based protein erasers (SNIPERs) as a potential therapeutic strategy. Eur. J. Med. Chem..

[402] Itoh Y., Ishikawa M., Naito M., Hashimoto Y. (2010). Protein knockdown using methyl bestatin-ligand hybrid molecules: Design and synthesis of inducers of ubiquitination-mediated degradation of cellular retinoic acid-binding proteins. J. Am. Chem. Soc..

[403] Ohoka N., Okuhira K., Ito M., Nagai K., Shibata N., Hattori T., Ujikawa O., Shimokawa K., Sano O., Koyama R. (2017). In Vivo Knockdown of Pathogenic Proteins via Specific and Nongenetic Inhibitor of Apoptosis Protein (IAP)-dependent Protein Erasers (SNIPERs). J Biol. Chem..

[404] Dueber E.C., Schoeffler A.J., Lingel A., Elliott J.M., Fedorova A.V., Giannetti A.M., Zobel K., Maurer B., Varfolomeev E., Wu P. (2011). Antagonists induce a conformational change in cIAP1 that promotes autoubiquitination. Science.

[405] Edmondson S.D., Yang B., Fallan C. (2019). Proteolysis targeting chimeras (PROTACs) in ‘beyond rule-of-five’ chemical space: Recent progress and future challenges. Bioorg. Med. Chem. Lett..

[406] Pop C., Timmer J., Sperandio S., Salvesen G.S. (2006). The apoptosome activates caspase-9 by dimerization. Mol. Cell.

[407] Hideshima T., Bradner J.E., Wong J., Chauhan D., Richardson P., Schreiber S.L., Anderson K.C. (2005). Small-molecule inhibition of proteasome and aggresome function induces synergistic antitumor activity in multiple myeloma. Proc. Natl. Acad. Sci. USA.

[408] Cenci S., Oliva L., Cerruti F., Milan E., Bianchi G., Raule M., Mezghrani A., Pasqualetto E., Sitia R., Cascio P. (2012). Pivotal Advance: Protein synthesis modulates responsiveness of differentiating and malignant plasma cells to proteasome inhibitors. J. Leukoc Biol..

[409] Fuchs D., Berges C., Opelz G., Daniel V., Naujokat C. (2008). Increased expression and altered subunit composition of proteasomes induced by continuous proteasome inhibition establish apoptosis resistance and hyperproliferation of Burkitt lymphoma cells. J. Cell Biochem..

[410] Leung-Hagesteijn C., Erdmann N., Cheung G., Keats J.J., Stewart A.K., Reece D.E., Chung K.C., Tiedemann R.E. (2015). Xbp1s-Negative Tumor B Cells and Pre-Plasmablasts Mediate Therapeutic Proteasome Inhibitor Resistance in Multiple Myeloma. Cancer Cell.

[411] Reimold A.M., Iwakoshi N.N., Manis J., Vallabhajosyula P., Szomolanyi-Tsuda E., Gravallese E.M., Friend D., Grusby M.J., Alt F., Glimcher L.H. (2001). Plasma cell differentiation requires the transcription factor XBP-1. Nature.

[412] Perez-Galan P., Mora-Jensen H., Weniger M.A., Shaffer A.L., Rizzatti E.G., Chapman C.M., Mo C.C., Stennett L.S., Rader C., Liu P. (2011). Bortezomib resistance in mantle cell lymphoma is associated with plasmacytic differentiation. Blood.

[413] Mani A., Gelmann E.P. (2005). The ubiquitin-proteasome pathway and its role in cancer. J. Clin. Oncol..

[414] Liu Y., Duan C., Zhang C. (2021). E3 Ubiquitin Ligase in Anticancer Drugdsla Resistance: Recent Advances and Future Potential. Front. Pharmacol..

[415] Um H.D. (2016). Bcl-2 family proteins as regulators of cancer cell invasion and metastasis: A review focusing on mitochondrial respiration and reactive oxygen species. Oncotarget.

[416] Tu H., Costa M. (2020). XIAP’s Profile in Human Cancer. Biomolecules.

[417] Ku B., Liang C., Jung J.U., Oh B.H. (2011). Evidence that inhibition of BAX activation by BCL-2 involves its tight and preferential interaction with the BH3 domain of BAX. Cell Res..

